# Providing Lesbian, Gay, Bisexual, Transgender, Nonbinary, and Queer Adolescents With Nurturance, Trustworthiness, and Safety: Protocol for Pilot Cluster Randomized Controlled Trial Design

**DOI:** 10.2196/55210

**Published:** 2024-03-19

**Authors:** Robert WS Coulter, Isabella Kaur Mahal, Clarisse A Lin, Shari Kessel Schneider, Aaryn S Mathias, Karuna Baral, Elizabeth Miller, Kaleab Z Abebe

**Affiliations:** 1 Department of Behavioral and Community Health Sciences School of Public Health University of Pittsburgh Pittsburgh, PA United States; 2 Department of Pediatrics School of Medicine University of Pittsburgh Pittsburgh, PA United States; 3 Division of Adolescent and Young Adult Medicine UPMC Children's Hospital of Pittsburgh Pittsburgh, PA United States; 4 Educational Development Center, Inc Waltham, MA United States; 5 Division of General Internal Medicine School of Medicine University of Pittsburgh Pittsburgh, PA United States

**Keywords:** sexual minority youths, gender minority youths, cluster randomized controlled trial, web-based behavior change intervention, high school staff

## Abstract

**Background:**

Sexual and gender minority youths (lesbian, gay, bisexual, transgender, nonbinary, and queer individuals) face elevated risks of substance use (eg, alcohol and tobacco) and mental health issues (eg, depressive symptoms and suicidality) compared to their cisgender heterosexual peers. These inequities are hypothesized to be reduced by building supportive high school environments via the training of school staff. An intervention that trains school staff to better understand and support sexual and gender minority youths and engage in positive bystander behaviors that protect them from bullying exposure may reduce disparities in drug and alcohol use among them. Experts, school staff, and sexual and gender minority youths developed Providing LGBTQ+ Adolescents with Nurturance, Trustworthiness, and Safety (PLANTS), a web-based intervention to train school staff on how to support, affirm, and protect sexual and gender minority youths.

**Objective:**

This paper describes the design of the PLANTS pilot trial primarily aimed at assessing its acceptability, usability, appropriateness, and feasibility. We hypothesize PLANTS will have high acceptability, usability, appropriateness, and feasibility as rated by the school staff. Secondary objectives focus on implementation, safety, and pre-post changes in high school staff outcomes, including self-efficacy and skills (eg, active-empathic listening and bullying intervention). Exploratory objectives focus on the impact of PLANTS on student health outcomes.

**Methods:**

In a 2-arm cluster randomized controlled trial, high schools in Massachusetts are allocated to PLANTS or an active comparator group (publicly available sexual and gender minority youths resources or training). High school staff complete pretest and posttest surveys containing validated scales. Primary outcomes are validated measures of acceptability, usability, appropriateness, and feasibility of the intervention completed by staff during posttest surveys. To test our primary hypotheses for each outcome, we will calculate means and 95% CIs and *P* values using 1-sample 2-sided *t* tests against a priori thresholds or benchmarks of success. Secondary outcomes include staff’s active-empathetic listening skills, self-efficacy for working with sexual and gender minority youths, bystander intervention behaviors for bullying and cyberbullying, and self-efficacy for PLANTS’ change objectives completed during pretest and posttest staff surveys. Staff can also complete a posttest interview guided by the Information-Motivation-Behavior model and Consolidated Framework for Implementation Research. Exploratory outcomes include student-level data collected via the 2021 and 2023 MetroWest Adolescent Health Surveys, a health behavior surveillance system in 30 Massachusetts schools.

**Results:**

School enrollment began in May 2023 and participant enrollment began in June 2023. Data collection is expected to be completed by February 2024.

**Conclusions:**

This pilot trial will yield important information about the PLANTS intervention and provide necessary information to conduct a fully powered trial of the efficacy of PLANTS for reducing the deleterious health inequities experienced by sexual and gender minority youths.

**Trial Registration:**

ClinicalTrials.gov NCT05897827; https://clinicaltrials.gov/study/NCT05897827

**International Registered Report Identifier (IRRID):**

DERR1-10.2196/55210

## Introduction

### Background

Sexual and gender minority youths (ie, adolescents who are lesbian, gay, bisexual, transgender, nonbinary, or queer [LGBTQ+]) are at significantly higher risk than their heterosexual peers for mental health problems and substance use [1-22]. For example, alcohol use is 123%-155% higher among sexual minority youths than among heterosexual youths and up to 250% higher among gender minority youths than among cisgender youths [2,5-7,22]. These substantial persistent health inequities make sexual and gender minority youths a priority population for interventions and were deemed so by national health agencies [3,23,24]. However, there are few efficacious substance use and mental health interventions for sexual and gender minority youths [3].

One way to reduce substance use and improve mental health for sexual and gender minority youths is to foster more supportive and inclusive high school environments by training school staff (eg, teachers, principals, nurses, and counselors) to effectively support and protect them. Sexual and gender minority youths who have support from adults at school, greater school connectedness, and lower bullying exposure also have reduced depressive symptoms, suicidality, and drug and alcohol use [5,25-30].

Unfortunately, sexual and gender minority youths are more likely than their heterosexual peers to lack supportive adults at school, have lower school connectedness, and be exposed to bullying [5,6,25,26,31-39]. An intervention that trains school staff to better understand and support sexual and gender minority youths and engage in positive bystander behaviors that protect them from bullying exposure may reduce health disparities among them. Despite many school staff having a strong desire to support sexual and gender minority youths [40], their primary barrier to supporting this population is a lack of training [40,41]. In 2014, 13% of teachers across the United States and 29% in Massachusetts received training on issues related to sexual and gender minority youths [41], highlighting the need for professional development training related to this population in schools. An intervention for training school staff is further warranted because the presence of gender-sexuality alliances and sexual and gender minority youth–inclusive school policies fail to eliminate health disparities among them [4,42-44].

Providing LGBTQ+ Adolescents with Nurturance, Trustworthiness, and Safety (PLANTS) is a new web-based training program for high school staff. This intervention was informed by the Information-Motivation-Behavior theory to target the skills, self-efficacy, knowledge, and outcome expectations of the high school staff. School staff and other collaborators invested in the well-being of sexual and gender minority youths assisted in developing PLANTS. PLANTS aims to train school staff to support, affirm, and protect sexual and gender minority youths, which is hypothesized to reduce bullying exposure, increase school support and connectedness, and mitigate health disparities among them [45].

Prior to testing efficacy, it is critical to ensure that PLANTS is acceptable to high school staff. This paper describes the design of the PLANTS pilot trial, which primarily tests the PLANTS intervention acceptability (perceptions that PLANTS is tolerable), usability (perceived extent to which PLANTS can be used effectively, efficiently, and satisfactorily), appropriateness (perceived fit and relevance of PLANTS), and feasibility (the extent to which the PLANTS intervention is successfully used and executed) as reported by high school staff. Using a cluster randomized design, this study will secondarily examine the implementation, safety, and pre-post changes in high school staff outcomes within the PLANTS arm and then compare them to an active comparator condition composed of publicly available resources. This study will also explore intervention effects on student-level behavioral health outcomes. The results from the PLANTS pilot trial will inform the development of a fully powered trial of the efficacy of PLANTS for improving health outcomes among sexual and gender minority youths.

### Objectives and Hypotheses

The primary objective of the PLANTS pilot trial is to assess the acceptability, usability, appropriateness, and feasibility of the intervention. Investigators expect that high school staff will rate the PLANTS intervention as having high acceptability, usability, appropriateness, and feasibility. Investigators’ benchmarks of success are averages of >3.75 out of 5 for acceptability, appropriateness, and feasibility and scores >75 out of 100 for usability.

The secondary objectives are to examine trial implementation, intervention demand, intervention safety, and pre-post changes in school staff outcomes within the PLANTS arm and then compare them to an active comparator condition. Investigators hypothesize the following results: school staff will have high participation rates in the study (≥50% consent); school staff will have a high retention rate for the follow-up survey (≥75%); high school staff in the PLANTS arm will have high intervention demand (≥75% adhere to PLANTS); high school staff in the PLANTS arm will have low adverse event prevalence (≤20% of PLANTS participants will report adverse events); high school staff participants in the PLANTS arm will report pre-post improvements in active-empathic listening, self-efficacy for supporting, affirming, and protecting sexual and gender minority youths; positive bystander intervention behaviors for bullying; and pre-post differences will be greater in the PLANTS arm than in the active comparator arm.

The exploratory objectives concern student-level health outcomes, including substance use, mental health, violence, and school experiences. Investigators hypothesize that sexual and gender minority youths will have greater increases in adult support at school and school connectedness and greater reductions in bullying exposure, depressive symptoms, suicidality, drug use, and alcohol use in PLANTS intervention schools versus active comparator schools, and the differences between sexual and gender minority youths and cisgender heterosexual youths in alcohol use, drug use, depressive symptoms, and suicidality will be more reduced in PLANTS intervention schools versus the active comparator schools.

## Methods

### Design

**Figure 1:**
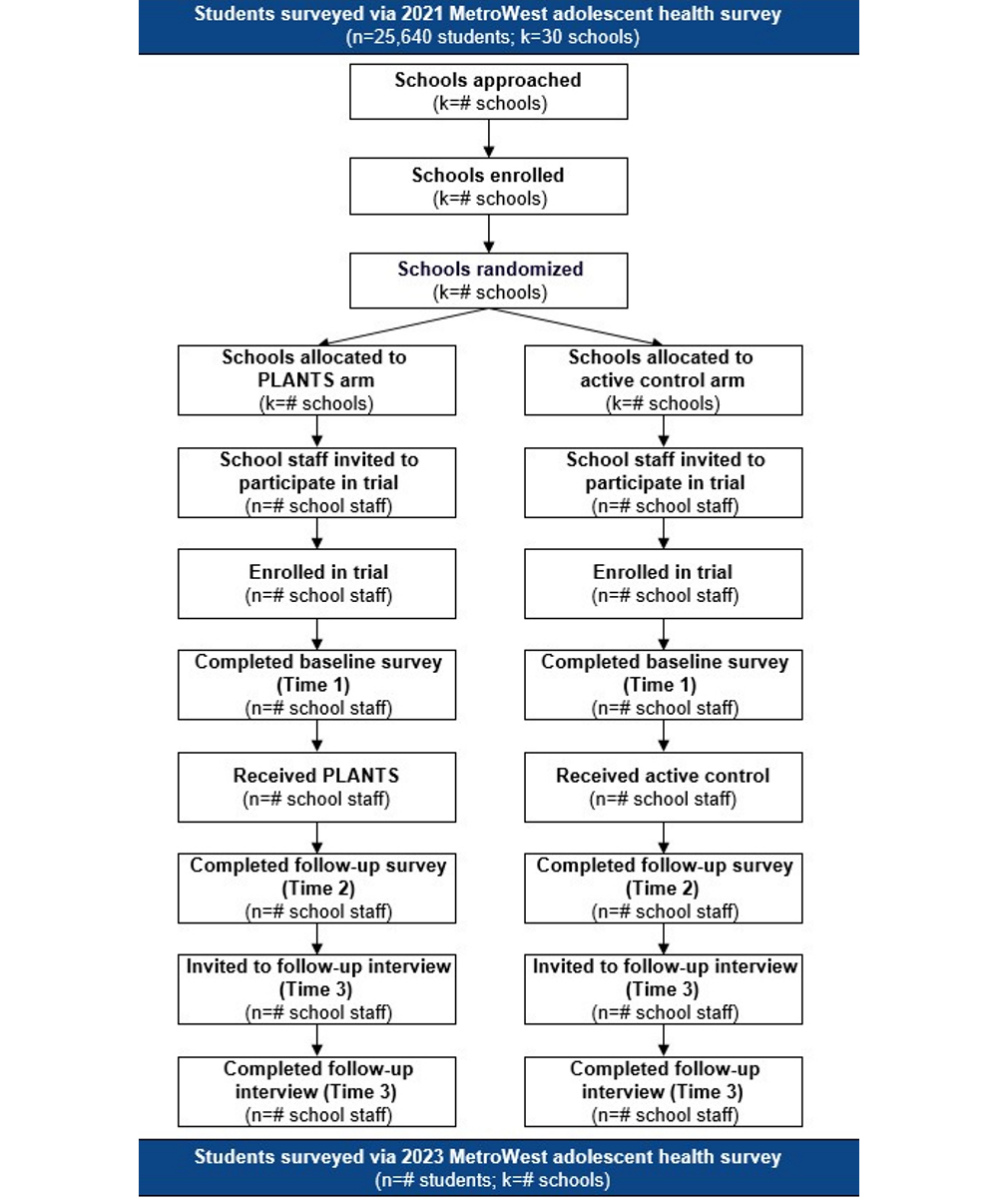
Study flow of the PLANTS pilot trial. PLANTS: Providing LGBTQ+ Adolescents with Nurturance, Trustworthiness, and Safety.

The PLANTS pilot trial is a cluster randomized controlled trial with 2 parallel groups and primary end points of PLANTS acceptability, usability, appropriateness, and feasibility among school staff. Such outcomes are aligned with pilot study best practices [46-49]. This unblinded study will randomly assign 4 schools in a 1:1 ratio to the intervention or comparator conditions. Importantly, investigators will analyze both student- and staff-level outcomes before and after the intervention. Figure 1 shows the study flow.

### Setting

This study will enroll high schools (grades 9-12) from the 30 schools participating in the MetroWest Adolescent Health Survey (MWAHS) located in and near the MetroWest Region outside Boston, Massachusetts. The PLANTS pilot trial leverages the strengths of the MWAHS research infrastructure, which has biennially collected health surveillance data from a census of students since 2006. To be eligible for the PLANTS pilot trial, a school must have participated in the 2021 MWAHS, plan to participate in the 2023 MWAHS, grant investigators permission to access their MWAHS data, be willing and able to provide email addresses of all school staff, and provide a site permission letter.

### Randomization

School-level randomization occurs after schools enroll in the study but before staff are enrolled. The investigators will use block randomization in a 1:1 ratio, stratified by larger schools (≥1000 students) versus smaller schools (<1000 students). The primary investigator will create the randomization files using the “ralloc” package in Stata (StataCorp). Trained study staff will allocate schools using the REDCap (Research Electronic Data Capture; Vanderbilt University) randomization module. Allocation will be concealed from school personnel.

### Study Populations, Sampling, Recruitment, and Data Collection

#### Students

To be eligible to participate in the MWAHS, students must be enrolled in grades 9-12 at a study school and be literate in English, Spanish, or Portuguese. Students are excluded if they provide an implausible pattern of responses via an evidence-informed algorithm that removes students with extreme responses.

Biennially, MWAHS collects student-level health surveillance data, similar to the Youth Risk Behavior Survey [50] except MWAHS data are collected from a census of students in each high school. The census-like sampling is a major strength of this study, providing a substantial sample of sexual and gender minority youths. Administered via the internet, the MWAHS is voluntary, anonymous, and data are linked at the school level across years (but cannot be linked at the student level). The baseline for intervention efficacy for students will be the fall 2021 MWAHS data, when 30 high schools participated, and 25,640 students completed surveys (83% of all students). The follow-up occurred in fall 2023.

#### Staff

To be eligible to participate in the PLANTS pilot trial, staff must be currently employed by an enrolled school, be 18 years or older, and consent to participate. Staff are excluded from participation if they do not interact directly with high school students at work.

At enrolled schools, all school staff will be invited to participate via email and advertisements sent by research staff and school administrators. Using REDCap, a personalized link to the screening survey will be provided to staff. If eligible, individuals will be given a digital informed consent form followed by a 30- to 45-minute baseline survey. Intervention and comparator conditions will be delivered to participants over 6 weeks. At the program’s conclusion, participants will be sent a follow-up survey. A subset of participants (n=20-30 in PLANTS and n=10-20 in e-Learning to Maximize Academic Inclusion of LGBTQ+ Students [EMAILS] arms) will then be invited for a follow-up interview to explore the implementation outcomes more deeply.

### Ethical Considerations

#### Staff

The Human Research Protection Office at the University of Pittsburgh approved the trial (STUDY23040142). Informed consent is obtained from staff participants. Consent forms describe in detail the study intervention, study procedures, foreseeable risks and discomforts, benefits to the participant, and contact information for the principal investigator. We requested and received a waiver for written consent for all staff participants because consent procedures are happening digitally, the study presents no more than minimal risk to participants, and written consent is not usually obtained for participation in a web-based program or interview outside of a research context.

Privacy and confidentiality protections are in place. For all identifiable data collected, we will remove identifiers and assign a unique study ID to protect the identity of the participant. Coded deidentified data and identifiable data will be stored in separate REDCap surveys and separate folders within a secure password-protected database and will be only accessible to select members of the research team.

To incentivize school staff to complete the baseline survey, we will provide US $20 to each participant and will conduct a drawing of an extra US $30 to 1 in 5 participants who take the survey within each school. To incentivize school staff to complete the follow-up survey, we will provide US $30 to each participant who completes the survey, and we will conduct a drawing of an extra US $40 to 1 in 5 participants who take the survey within each school. For staff who complete the follow-up interview, we will provide a US $50 incentive as a thank you for their time.

#### Students

The Education Development Center’s institutional review board–approved the MWAHS. Parents or guardians are provided the opportunity to opt their child out of the survey (ie, passive consent), and students provide assent to participate. Assent forms describe in detail the study procedures, foreseeable risks and discomforts, benefits to the participant, and contact information for the principal investigator. Data are collected anonymously, preserving student privacy and confidentiality. No incentives are provided to students for participation.

### PLANTS Intervention

**Figure 2:**
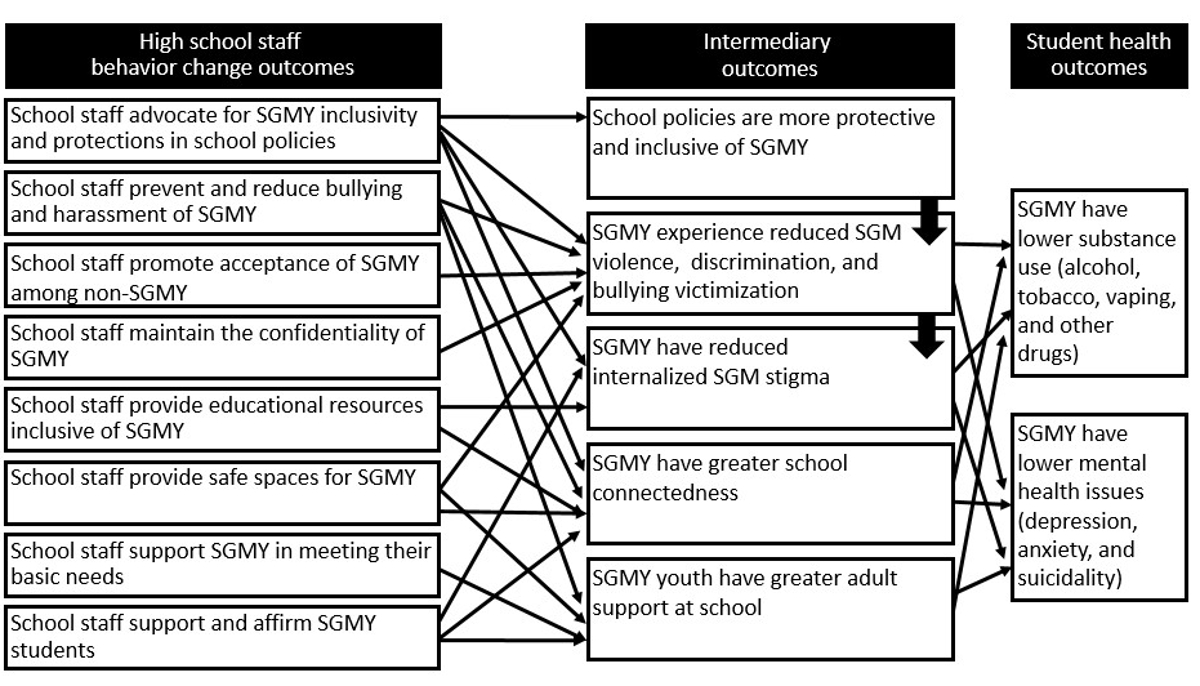
Behavior change model with high school staff behavior outcomes and student health outcomes. SGM: sexual and gender minority; SGMY: sexual and gender minority youths.

The PLANTS intervention is a web-based training program for high school staff. Figure 2 illustrates the PLANTS Behavior Change Model. Staff behavior change outcomes target evidence-based intermediary outcomes rooted in theories of minority health and general adolescent psychosocial health models. In turn, these intermediary outcomes are associated with reduced substance use and mental health issues. The behavior change outcomes are that PLANTS uses asynchronous and synchronous web-based activities to achieve the behavioral change outcomes via targeting the skills, self-efficacy, knowledge, and outcome expectations of the school staff based on Information-Motivation-Behavior theory. PLANTS has 3 primary modules: trustworthiness, safety, and nurturance. Asynchronous activities include voiceover presentations, podcasts with student and staff stories based on research [51], activities, and downloadable resources for future reference. Synchronous activities include three 1.5-hour live Zoom events; each moderated by a trained interventionist and tailored to the needs of participants. The modules were developed by the research team, including undergraduate and graduate students with a variety of academic backgrounds, in partnership with high school staff and other professionals who specialize in LGBTQ+ youth or education. PLANTS is delivered using Canvas Learning management software (Instructure).

### Active Comparator: EMAILS

Given the urgent need to support sexual and gender minority youths coupled with the dearth of evidence-based interventions for reducing alcohol and drug use in this population [3], choosing a comparator was difficult. Pragmatically, staff may search the internet to identify training opportunities. Thus, the active comparator, EMAILS, is an email-based intervention comprised of free existing web-based resources for supporting, affirming, and protecting sexual and gender minority youths. Informed by the Information-Motivation-Behavior theory, EMAILS has materials from Adagio Health, the Gay, Lesbian, & Straight Education Network, the American Psychological Association, and the Human Rights Campaign, which include self-paced training modules, YouTube videos, and PowerPoints. There is no direct human interaction in this intervention other than email. EMAILS is 3 hours long and is delivered in 3 modules as in PLANTS. Investigators will monitor active comparator compliance and fidelity by disseminating materials in Qualtrics, which allows for personalized link tracking and short end-of-module questionnaires about uptake or completion.

### Outcomes

#### Primary Outcomes

The primary outcomes are intervention acceptability, usability, appropriateness, and feasibility as reported by school staff. At the follow-up survey, these are measured via scales with strong psychometric properties such as the acceptability of intervention measure (AIM), System Usability Scale (SUS), intervention appropriateness measure (IAM), and feasibility of intervention measure (FIM) [52,53]. AIM, IAM, and FIM each have 4 items with 5-point Likert scale response options. This instrument can be found in Table S1 in Multimedia Appendix 1. Investigators will calculate mean scores (range: 1-5). The SUS has 10 items with 5-point Likert scale response options. Investigators will calculate scores as recommended for the total scale (range: 0-100) [54]. The same outcomes are assessed about EMAILS among comparator participants, but these are not primary outcomes.

#### Secondary Outcomes

##### Trial Implementation

To assess the success of the pilot trial in reaching an adequate number of school staff, investigators assess the overall trial participation rate (number of people enrolled divided by the number of people invited to participate) and the follow-up survey retention rate (the number of people who take the follow-up survey divided by the number of people enrolled).

##### PLANTS Intervention Demand

To assess the school staff’s demand for PLANTS, investigators assess PLANTS adherence, which is a composite variable ranging from 0% to 100%, comprised of 55% for module completion (based on the number of completed items divided by the total number of items offered) and 45% for Live Zoom Event attendance (where each event is 15%). These proportions are based on approximate time allocations.

##### Safety

Investigators assess a myriad of safety outcomes in follow-up surveys, including contact from parents or guardians because there was too much LGBTQ+ inclusivity in the school, contact from people who were upset, the school being attacked, the school board getting upset or concerned, and LGBTQ+ censorship at the school. Response options include the frequency of each event occurrence. Investigators also assess participants’ emotional discomfort with the courses using a 4-point Likert scale. For affirmative responses, open-ended textboxes are provided to describe the safety-related events. Investigators also track the presence or severity of adverse events and unanticipated problems.

##### Self-Efficacy for Working With Sexual and Gender Minority Youths

Investigators assess participants’ perceived abilities for working with LGBTQ high school students using 9 items adapted from the Gay Affirmative Practice Scale [55]. Originally for social work practitioners, investigators replaced therapy-oriented words with school-oriented words (eg, “students” instead of “clients”).

Example items include “I am able to help LGBTQ+ students develop positive identities as LGBTQ+ individuals” and “I am able to challenge misinformation about LGBTQ+ individuals in the classroom.” Response options include a 5-point Likert scale. Investigators will calculate the mean score. In a prior study, the Cronbach α is 0.90 [56].

##### Active-Empathic Listening

Investigators measure the valid and reliable Active-Empathic Listening Scale containing 11 items [57]. This scale has 3 domains: sensing (4 items), processing (3 items), and responding (4 items). Response options include a 7-point Likert scale. Investigators will calculate the mean score for the total scale. Prior research showed a Cronbach score of α=0.88-0.90 [56].

##### Bystander Intervention Behaviors for Bullying and Cyberbullying

Two multidimensional scales (Teacher Bystander Intervention Model in Traditional Bullying and Teacher Bystander Intervention Model in Cyberbullying [58,59]) measure 5 subscales of bystander behaviors for bullying and cyberbullying: noticing the event (3 items), interpreting the event as an emergency (3 items), accepting responsibility to help (3 items), knowing how to help (3 items), and implementing intervention decision (4 items). The psychometric properties of these subscales are acceptable (Cronbach α=0.57-0.88). Investigators will calculate average subscale scores.

##### Self-Efficacy of PLANTS’ Change Objectives

Given the limited research on validated scales of behavior change pertaining to LGBTQ+ inclusive practices in schools, investigators developed items pertaining directly to the self-efficacy change objectives in PLANTS. There are 50 total items across the following domains: provide interpersonal support and affirmation to sexual and gender minority youths; provide educational resources that are inclusive of this population; provide safe spaces for them; promote the acceptance of this population among cisgender heterosexual youths; prevent and reduce bullying, cyberbullying, and harassment of this population; evaluate and advocate for their inclusivity and protections in school policies; and maintain the confidentiality of sexual and gender minority youths.

#### Exploratory Outcomes

Student-level outcomes will be explored using the MWAHS data. Measures are described in Table S2 in Multimedia Appendix 1, and most have strong test-retest reliability and internal consistency.

#### Follow-Up Interview Questions

The purpose of the follow-up interview is to better understand trial and intervention implementation. Interviews are guided by the CFIR (Consolidated Framework for Intervention Research) [60]. Question domains include intervention characteristics (relative advantage, adaptability, and design quality), outer setting (external policies and incentives), inner setting (structural characteristics, networks, communication, culture, implementation climate, compatibility, relative priority, and leadership engagement), characteristics of individuals (beliefs about the intervention and self-efficacy), and process (opinion leaders). Interview questions are based on the publicly provided CFIR interview questions [60].

### Demographics and Potential Confounders

Staff and student surveys assess many potential confounders. Table S3 in Multimedia Appendix 1 contains measurement details. School-level data will be collected from the Massachusetts Department of Elementary and Secondary Education public website.

### Analyses

#### General Approach

Investigators will calculate baseline descriptive statistics for each study arm and test for differences in potential confounders between intervention and comparator arms using baseline student-, staff-, and school-level data with Rao-Scott chi-square tests and linear mixed models accounting for school clustering. Secondary analyses will adjust for imbalances between arms.

For validated scales, investigators will report internal consistency via Cronbach α. For newly created items, investigators will conduct exploratory factor analyses to examine the dimensions of outcomes using baseline surveys. Investigators will use the most recent version of Stata, 2-tailed tests, and α=.05.

Investigators will also conduct bias assessments. Selection bias assessment will compare participants’ demographics to publicly available school-level data. The attrition bias assessment will compare staff respondents who completed follow-up surveys versus those who did not by baseline demographics and outcomes. Investigators will report significant differences as potential validity threats.

#### Primary Outcome Analyses

To answer the primary research questions, investigators will use best practices for pilot or feasibility studies [46-49]. Investigators will analyze the primary outcomes using descriptive statistics [46-49] and will not correct for multiple tests [46-49]. Among people in the PLANTS arm, investigators will calculate means and 95% CIs for participants’ responses to the FIM, AIM, IAM, and SUS [53] and *P* values using 1 sample two-sided *t* tests against a priori thresholds.

#### Secondary Outcome Analyses

For trial implementation outcomes, investigators will calculate the participation and retention rates with a percentage and 95% CIs in the overall study sample. For PLANTS intervention demand, investigators will calculate average adherence with a percentage and a 95% CI among participants in the PLANTS study arm.

For safety outcomes, investigators will estimate the prevalence of adverse events reported by school staff at any time after intervention or comparator deployment. Investigators will report by study arm: the overall frequency of adverse events; the frequency of each type; and the frequency and percentage of school staff reporting adverse events.

To examine the pre-post changes in high school staff outcomes, investigators will first use descriptive statistics, such as means and percentages at each timepoint within arms. To test for within-arm statistical significance, investigators will use linear mixed models for continuous outcomes and generalized linear mixed models for binary outcomes, which account for within-school and within-person clustering using random effects. Investigators will estimate the intraclass correlations for within-school and within-person effects. These models will adjust for school size (a priori design variable).

Subsequently, investigators will compare pre-post changes between arms using regression models that include a fixed term for school size (a priori design variable), intervention group (intervention or comparator), time (baseline or follow-up), and the interaction of the intervention group×time (variable of interest for between-arm differences in pre-post changes). The intervention effects on secondary outcomes will be primarily based on intent-to-treat (ITT) estimates. Investigators will estimate as-treated and per-protocol effects in secondary models. If there are differences in potential confounders by intervention group, investigators will adjust for them in secondary multivariable analyses.

#### Exploratory Outcome Analyses

To explore the intervention effects among sexual and gender minority youths (within-group analyses), investigators will conduct ITT analyses using linear mixed models or generalized linear models accounting for within-school clustering effects using random effects. Investigators will restrict the sample to participants who reported a sexual minority identity or gender minority identity. Regression models will include a fixed term for school size (a priori design variable), intervention group (intervention or comparator), time (baseline or follow-up), and the interaction of intervention group×time (variable of interest). First, investigators will estimate ITT effects. Second, because subsetting a randomized sample may lead to naturally imbalanced arms, investigators will adjust for any imbalanced confounders.

To explore the intervention effects on inequities among sexual and gender minority youths (between-group analyses), investigators will use mixed models like previously described, except investigators who will include all student data in these analyses, including cisgender heterosexual youths, to assess reductions in inequities. Regression models will include fixed terms for the intervention group (PLANTS or EMAILS), time (baseline or follow-up), all 2-way interactions between the intervention group, time, and sexual and gender minority youths, and the 3-way interaction of the intervention group×time×sexual and gender minority youths (exploratory variable of interest for this hypothesis). Investigators will primarily explore the ITT effects.

#### Qualitative Analyses

Investigators will transcribe, deidentify, and check the quality of all data [61-64]. Investigators will perform use CFIR as a guiding framework. Two trained qualitative coders from our research team will independently read interviews and compare coding until they agree. Once the coders agree all major codes have been identified, they will create a final codebook with definitions, rules, and examples for each code [63,64]. Two coders will then recode all data using the final codes. Investigators will calculate inter-rater reliability (Kappa statistic) to examine code application between coders [65]. Coders will discuss any discrepancies until they reach an agreement; the principal investigator (RWSC) will resolve disagreements [63,64]. Investigators will use either a qualitative descriptive coding approach [66] (describing and counting code applications) or axial coding [67] (combining inductive codes into broader categories to define emerging patterns or themes). Investigators will identify and interpret patterns using thematic analysis [68].

### Sample Size

Investigators calculated statistical power based on the primary outcomes, a 5% error rate, and best practices for feasibility studies. The median number of teachers at each MetroWest region high school is 86. With 4 schools, investigators anticipate inviting a total of ≥344 school staff (including teachers and other school staff with direct contact with students) to participate in the pilot study. Assuming 50% agree to participate, 50% of participants are in the PLANTS study arm, and 75% of PLANTS participants complete the follow-up survey (reduced n=65), investigators can estimate 95% CI widths ≤0.33 for AIM and IAM, and ≤10.1 for SUS. Such precision levels are sufficient. For qualitative interviews, investigators aim to interview people with a diversity of intervention fidelity, acceptability, usability, feasibility, and appropriateness. This is an exploratory interview study in nature, so idea generation and exploration are the goal, not thematic saturation. Investigators aim to interview PLANTS (n=20-30) and EMAILS (n=10-20) participants, and these sample sizes will provide ample information about the CFIR domains.

## Results

School enrollment began in May 2023 and participant enrollment began in June 2023. Data collection is expected to be completed by January 2024. As of December 4, 2023, a total of 99 school staff enrolled in the study. Data collection is expected to be completed in January 2024.

## Discussion

### Principal Findings

This pilot trial rigorously evaluates the acceptability, usability, appropriateness, and feasibility of PLANTS, a web-delivered intervention aimed at improving school staff’s skills, self-efficacy, knowledge, and outcome expectations for working with sexual and gender minority youths. Schools provide an ideal setting for interventions specific to sexual and gender minority youths for health disparities. High school students spend ~1195 hours per year in school [69], and sexual and gender minority youths regularly interact with adults who are professionally bound by certifying bodies to support the needs of students, including this population [70-73]. Implementing interventions in schools is challenging because of schools’ limited resources, increasing demands placed on teachers, and difficulty in acquiring school buy-in. By using an economical and easily implementable web-based intervention, and by developing interventions and implementation strategies in collaboration with school personnel, these barriers may be overcome. PLANTS meets each of these criteria. A strength of this study is how it is embedded within the existing surveillance infrastructure. MWAHS conducts a census of students and has high student participation rates, minimizing biases common in convenience samples of sexual and gender minority youths.

### Limitations

The primary limitation is generalizability because the study is solely in Massachusetts, a predominantly liberal US state. Selection bias could be present, for example, if school staff with the greatest stigmatizing attitudes toward sexual and gender minority youths do not participate. Investigators will examine if attitudes toward sexual and gender minority youths are associated with retention or attrition. Despite using numerous validated measures, the reliance on self-reported measures for both staff and students can be seen as a limitation. Because there is concern that school staff may be dishonest, investigators include a measure of social desirability bias [74]. Investigators will control for social desirability in analyses if it is high or if it is unevenly distributed among intervention versus comparator schools. For the exploratory student-level outcomes, another limitation is the lack of assessing individual-level change in student outcomes, since MWAHS data are collected biennially and anonymously. Investigators minimize historical and maturation biases by comparing youths in intervention schools to their same-aged peers in comparator schools across the same time periods while also assessing school-level policy and programmatic changes via surveys.

### Conclusions

This study will rigorously test the hypothesis that PLANTS will be rated highly acceptable, usable, appropriate, and feasible by high school staff. PLANTS is hypothesized to be more efficacious for improving staff’s support of sexual and gender minority youths and therefore reducing health inequities in this population than the active comparator. The results from this pilot trial will inform a fully powered trial of the efficacy of PLANTS for fostering health equity in sexual and gender minority youths.

## Appendix

Multimedia Appendix 1 Survey items for primary outcomes, secondary outcomes, exploratory outcomes, demographics, and confounders.

Multimedia Appendix 2 Peer-reviewer report from the National Institute on Alcohol Abuse and Alcoholism.

## Abbreviations

AIM: Acceptability of Intervention Measure
CFIR: Consolidated Framework for Intervention Research
EMAILS: e-Learning to Maximize Academic Inclusion of LGBTQ+ Students
FIM: feasibility of intervention measure
IAM: intervention appropriateness measure
ITT: intent-to-treat
LGBTQ: Lesbian, Gay, Bisexual, Transgender, Nonbinary, and Queer
MWAHS: MetroWest Adolescent Health Survey
REDCap: Research Electronic Data Capture
PLANTS: Providing LGBTQ+ Adolescents with Nurturance, Trustworthiness, and Safety
SUS: System Usability Scale

## Data Availability

The data sets generated and analyzed during this study are available from the corresponding author on reasonable request.
