# Supplementary material for: Providing Lesbian, Gay, Bisexual, Transgender, Nonbinary, and Queer Adolescents With Nurturance, Trustworthiness, and Safety: Protocol for Pilot Cluster Randomized Controlled Trial Design
Source: JMIR Res Protoc. 2024 Mar 19;13:e55210. doi: 10.2196/55210 (PMC10988370; doi:10.2196/55210)
Supplement: Multimedia Appendix 1 [file resprot_v13i1e55210_app1.pdf]

	LGBTQ+ students with a safe space? Politely correct people who use students' incorrect names? Politely correct people who use students' incorrect pronouns? Teach students about LGBTQ+ terminology in a positive manner? Provide positive verbal reinforcement when non-LGBTQ+ students do something LGBTQ-inclusive? Define anti-LGBTQ+ bullying and harassment? Implement classroom guidelines and meetings? Intervene in anti-LGBTQ+ bullying and harassment? Convene an anti-LGBTQ+ bullying task force when necessary? Follow-up with victims and bystanders of anti-LGBTQ+ bullying? Use non-confrontational ways to engage with perpetrators of anti-LGBTQ+ bullying? Enforce clear consequences for people who bully others? Explain to students why anti-LGBTQ+ bullying is harmful and wrong? Advocate for evidence-based bullying prevention programming? Obtain LGBTQ+ students' feedback about the inclusivity and protections for LGBTQ+ students in existing school policies? Amend/dismantle school policies and practices that negatively affect LGBTQ+ students? Evaluate how inclusive and protective your school policies are for LGBTQ+ students and staff? Share statistics to advocate for LGBTQ+ inclusivity and protections in school policies? Share personal stories to advocate for LGBTQ+ inclusivity and protections in school policies? Use required methods (e.g., school board propositions) to create/amend school policy? Examine how well your school's LGBTQ+ related policies align with local, state, and federal school laws and policies? Keep students' sexual orientations confidential? Keep students' gender identities confidential? Make students aware of mandatory reporting guidelines? Assess students' safety concerns surrounding disclosure of their LGBTQ+ identity? Establish or amend school policies that protect the confidentiality of students' sexual orientations and gender identities?		
Teacher Bystander Intervention Model in Traditional Bullying	Traditional Bullying Definition: intentional and repeated harm inflicted through non-electronic means; consisting of three types: physical (e.g. kicking), verbal (e.g. calling someone names), and relational (e.g. leaving someone out on purpose). 1) Traditional bullying is a problem at this school. 2) I am aware that students at my school are traditionally bullied. 3) I have seen other students being traditionally bullied at my school this year. 4) It is evident to me that someone who is being traditionally bullied needs help. 5) Inappropriate comments can hurt someone's feelings, even if the person making the comment says they are joking. 6) I think traditional bullying is harmful and damaging to others. 7) I feel personally responsible to intervene and assist in resolving traditional bullying incidents. 8) I believe it is my responsibility to try and stop events of traditional bullying. 9) I believe that my actions can help to reduce traditional bullying. 10) I have the skills to support a student who is being treated disrespectfully. 11) I know what to say to	5-point Likert-type scale from "Strongly disagree" to "Strongly agree"	Mean score for each subscale

	get a student to stop traditionally bullying another student. 12) I can help get someone out of a situation where they are being traditionally bullied. 13) I would tell a group of students to stop using inappropriate language or behaviors if I see or hear them. 14) I would say something to a student who is acting mean or disrespectful to a more vulnerable student. 15) I would tell a student to stop using put-downs when talking about someone else. 16) If I saw a student I did not know very well being traditionally bullied at school, I would help get them out of the situation.		
Teacher Bystander Intervention Model in Cyberbullying	Cyberbullying definition: willful and repeated harm inflicted through the use of computers, cell phones, and other electronic devices. 1) Cyberbullying is a problem at this school. 2) I am aware that students at my school are cyberbullied. 3) I have witnessed events of cyberbullying at my school this year. 4) It is evident to me that someone who is being cyberbullied needs help. 5) Inappropriate comments online can hurt someone's feelings, even if the person making the comments says they are joking. 6) I think cyberbullying is hurtful and damaging to others. 7) I feel personally responsible to intervene and assist in resolving cyberbullying incidents. 8) I believe it is my responsibility to try and stop events of cyberbullying. 9) I believe that my actions can help to reduce cyberbullying. 10) I have the skills to support a student who is being treated disrespectfully online. 11) I know what to say to get a student to stop cyberbullying another student. 12) I can help get someone out of a situation where they are being cyberbullied. 13) I would tell a group of students to stop using inappropriate language or behaviors online. 14) I would say something to a student who is acting mean or disrespectful online to a more vulnerable student. 15) I would tell a student to stop using put-downs when talking about someone else online. 16) If a student I did not know very well was being bullied online, I would help get them out of the situation.	5-point Likert-type scale from "Strongly disagree" to "Strongly agree"	Mean score for each subscale
**SAFETY OUTCOMES**			
Emotional Discomfort with PLANTS	We will now ask some questions related to issues you may have encountered while completing/after completing the online program. 1) How uncomfortable were you while participating in the course?	4-point Likert-type scale from "Not at all uncomfortable" to "Very uncomfortable"	1) Any vs. none 2) Average score
General Backlash	Since you took your last survey, how many times were you contacted by people who were upset because you supported LGBTQ+ students?	Once, Twice, 3-9 times, 10 or more times	1) Any vs. none 2) Average frequency
Parent Backlash	Since you took your last survey, how many times were you contacted by parents/guardians because there was too much LGBTQ+ inclusivity in your school?	Once, Twice, 3-9 times, 10 or more times	1) Any vs. none 2) Average frequency

School Attacked	Since you took your last survey, how many times was your school attacked for supporting LGBTQ+ youth?	Once, Twice, 3-9 times, 10 or more times	1) Any vs. none<br>2) Average frequency
School Board Backlash	Since you took your last survey, how many times did your school board get upset or concerned about staff supporting LGBTQ+ youth?	Once, Twice, 3-9 times, 10 or more times	1) Any vs. none<br>2) Average frequency
LGBTQ+ Censorship	Since you took your last survey, how many times did LGBTQ+ censorship happen in your school?	Once, Twice, 3-9 times, 10 or more times	1) Any vs. none<br>2) Average frequency
Suspension or Removal from Employment	Since you took your last survey, how many times did you receive negative consequences from your employer about supporting LGBTQ+ youth?	Once, Twice, 3-9 times, 10 or more times	1) Any vs. none<br>2) Average frequency

Table S2. This table shows the exploratory outcomes based on student responses about health behaviors from the MetroWest Adolescent Health Survey.

Construct	Items assessed in 2021	Items assessed in 2023	Coding
**STUDENTS' PERCEPTIONS OF SCHOOL-BASED ADULT BEHAVIORS**			
Foster caring relationships	A 3-item scale: 1) At my school, there is a teacher or some other adult who really cares about me. 2) At my school, there is a teacher or some other adult who tells me when I do a good job. 3) At my school, there is a teacher or some other adult who notices when I'm not there. Not at all true, A little true, Pretty true, Very true	A 3-item scale: 1) At my school, there is a teacher or some other adult who really cares about me. 2) At my school, there is a teacher or some other adult who tells me when I do a good job. 3) At my school, there is a teacher or some other adult who notices when I'm not there. Not at all true, A little true, Pretty true, Very true	Mean score
Set high expectations	A 3-item scale: 1) At my school, there is a teacher or some other adult who always wants me to do my best. 2) At my school, there is a teacher or some other adult who listens to me when I have something to say. 3) At my school, there is a teacher or some other adult who believes that I will be a success. Not at all true, A little true, Pretty true, Very true	A 3-item scale: 1) At my school, there is a teacher or some other adult who always wants me to do my best. 2) At my school, there is a teacher or some other adult who listens to me when I have something to say. 3) At my school, there is a teacher or some other adult who believes that I will be a success. Not at all true, A little true, Pretty true, Very true	Mean score
Notice when students are in distress	How likely is it that teachers or other adults in your school notice when students are in distress (experiencing extreme anxiety, sorrow, or emotional pain)? Very unlikely, Unlikely, Neither, Likely, Very likely	How likely is it that teachers or other adults in your school notice when students are in distress (experiencing extreme anxiety, sorrow, or emotional pain)? Very unlikely, Unlikely, Neither, Likely, Very likely	Mean score
Understand how being in distress can affect a student's behavior	How likely is it that teachers or other adults in your school understand how being in distress can affect a student's behavior in class? Very unlikely, Unlikely, Neither, Likely, Very likely	How likely is it that teachers or other adults in your school understand how being in distress can affect a student's behavior in class? Very unlikely, Unlikely, Neither, Likely, Very likely	Mean score
Help students who are in distress get help and support	How likely is it that teachers or other adults in your school help students who are in distress get help and support at school? Very unlikely, Unlikely, Neither, Likely, Very likely	How likely is it that teachers or other adults in your school help students who are in distress get help and support at school? Very unlikely, Unlikely, Neither, Likely, Very likely	Mean score
**STUDENTS' CONNECTEDNESS AND INCLUSION AT SCHOOL**			
School connectedness	A 5-item scale: Do you agree or disagree with these statements about school? 1) I feel close to people at this school. 2) I feel happy to be at this school. 3) I feel like I am part of this school. 4) The teachers at this school treat students fairly. 5) I feel safe in this school. Strongly agree, Agree, Neither agree nor disagree, Disagree, Strongly disagree	A 5-item scale: Do you agree or disagree with these statements about school? 1) I feel close to people at this school. 2) I feel happy to be at this school. 3) I feel like I am part of this school. 4) The teachers at this school treat students fairly. 5) I feel safe in this school. Strongly agree, Agree, Neither agree nor disagree, Disagree, Strongly disagree	Mean score

Meaningful participation at school	A 3-item scale: Do you agree or disagree with the following statements? 1) At this school, students work on listening to others to understand what they are trying to say. 2) At this school, all students are treated equally. 3) At this school, students show respect for each other. Strongly agree, Agree, Neither agree nor disagree, Disagree, Strongly disagree	A 3-item scale: Do you agree or disagree with the following statements? 1) At this school, students work on listening to others to understand what they are trying to say. 2) At this school, all students are treated equally. 3) At this school, students show respect for each other. Strongly agree, Agree, Neither agree nor disagree, Disagree, Strongly disagree	Mean score
Feel they are part of a group of friends	How often do you feel like you are part of a group of friends? Never, Rarely, Sometimes, Often, Very often	How often do you feel like you are part of a group of friends? Never, Rarely, Sometimes, Often, Very often	Mean score
Has a lot in common with people around them	How often do you feel like you have a lot in common with the people around you? Never, Rarely, Sometimes, Often, Very often	How often do you feel like you have a lot in common with the people around you? Never, Rarely, Sometimes, Often, Very often	Mean score
Feel understood	How often do you feel like there are people who really know you and understand you? Never, Rarely, Sometimes, Often, Very often	How often do you feel like there are people who really know you and understand you? Never, Rarely, Sometimes, Often, Very often	Mean score
**STUDENTS' EXPERIENCES OF VIOLENCE AND DISCRIMINATION**			
Bullied at school	During the past 12 months, how many times have you been bullied on school property? 0 times, 1 time, 2 or 3 times, 4 or 5 times, 6 or 7 times, 8 or 9 times, 10 or 11 times, 12 or more times	During the past 12 months, how many times have you been bullied on school property? 0 times, 1 time, 2 or 3 times, 4 or 5 times, 6 or 7 times, 8 or 9 times, 10 or 11 times, 12 or more times	Any vs. none
Was in a physical fight at school	During the past 12 months, how many times were you in a physical fight on school property? 0 times, 1 time, 2 or 3 times, 4 or 5 times, 6 or more times	During the past 12 months, how many times were you in a physical fight on school property? 0 times, 1 time, 2 or 3 times, 4 or 5 times, 6 or more times	Any vs. none
Threatened or injured with weapon at school	During the past 12 months, how many times has someone threatened or injured you with a weapon such as a gun, knife, or club on school property? 0 times, 1 time, 2 or 3 times, 4 or 5 times, 6 or more times	During the past 12 months, how many times has someone threatened or injured you with a weapon such as a gun, knife, or club on school property? 0 times, 1 time, 2 or 3 times, 4 or 5 times, 6 or more times	Any vs. none
Social exclusion	How often do you feel left out and excluded by others? Never, Rarely, Sometimes, Often, Very often	How often do you feel left out and excluded by others? Never, Rarely, Sometimes, Often, Very often	Mean score
Sexuality-based harassment at school	During the past 12 months, how many times have you been bullied or called names, teased, made fun of, embarrassed, or threatened by other students on school property for the following reasons: because of your sexual identity or orientation (such as being gay, lesbian,	During the past 12 months, how many times have you been bullied or called names, teased, made fun of, embarrassed, or threatened by other students on school property for the following reason: because of your sexual identity or orientation (such as being gay, lesbian,	Any vs. none

	or bisexual) or what others think it is? Never, once, 2 or more times	or bisexual) or what others think it is? Never, once, 2 or more times	
Gender-based harassment at school	During the past 12 months, how many times have you been bullied or called names, teased, made fun of, embarrassed, or threatened by other students on school property for the following reasons: because of your gender? Never, once, 2 or more times	During the past 12 months, how many times have you been bullied or called names, teased, made fun of, embarrassed, or threatened by other students on school property for the following reasons: because of your gender? Never, once, 2 or more times	Any vs. none
Cyberbullied	During the past 12 months, how many times has someone used the internet, social media, cell phone, or other electronic device to bully, tease, threaten, or spread rumors about you? 0 times, 1 time, 2 or 3 times, 4 or 5 times, 6 or 7 times, 8 or 9 times, 10 or 11 times, 12 or more times	During the past 12 months, how many times has someone used the internet, social media, cell phone, or other electronic device to bully, tease, threaten, or spread rumors about you? 0 times, 1 time, 2 or 3 times, 4 or 5 times, 6 or 7 times, 8 or 9 times, 10 or 11 times, 12 or more times	Any vs. none
Sexuality-based harassment in digital environment	During the past 12 months, how many times has someone used the internet, social media, cell phone, or other electronic device to bully, tease, threaten, or spread rumors about you for the following reasons: because of your sexual identity or orientation (such as being gay, lesbian, or bisexual) or what others think it is? Never, once, 2 or more times	During the past 12 months, how many times has someone used the internet, social media, cell phone, or other electronic device to bully, tease, threaten, or spread rumors about you for the following reasons: because of your sexual identity or orientation (such as being gay, lesbian, or bisexual) or what others think it is? Never, once, 2 or more times	Any vs. none
Gender-based harassment in digital environment	During the past 12 months, how many times has someone used the internet, social media, cell phone, or other electronic device to bully, tease, threaten, or spread rumors about you for the following reasons: because of your gender? Never, once, 2 or more times	During the past 12 months, how many times has someone used the internet, social media, cell phone, or other electronic device to bully, tease, threaten, or spread rumors about you for the following reasons: because of your gender? Never, once, 2 or more times	Any vs. none
Worried or stressed about LGBTQ+ discrimination	How often do you worry or feel stressed about discrimination against LGBTQ people in your community (LGBTQ means lesbian, gay, bisexual, transgender, queer, or questioning)? Never, rarely, sometimes, often, very often	How often do you worry or feel stressed about discrimination against LGBTQ people in your community (LGBTQ means lesbian, gay, bisexual, transgender, queer, or questioning)? Never, rarely, sometimes, often, very often	Mean score
Did not go to school because they felt unsafe	During the past 30 days, on how many days did you not go to school because you felt you would be unsafe due to violence at school or on your way to or from school? 0 days, 1 day, 2 or 3 days, 4 or 5 days, 6 or more days	During the past 30 days, on how many days did you not go to school because you felt you would be unsafe due to violence at school or on your way to or from school? 0 days, 1 day, 2 or 3 days, 4 or 5 days, 6 or more days	Aby vs. none
**SUBSTANCE USE**			
Alcohol use	During the past 30 days, on how many days did you have at least one drink of alcohol? 0 days, 1 or 2 days, 3	During the past 30 days, on how many days did you have at least one drink of alcohol? 0 days, 1 or 2 days, 3	Any vs. none

	to 5 days, 6 to 9 days, 10 to 19 days, 20 to 29 days, All 30 days	to 5 days, 6 to 9 days, 10 to 19 days, 20 to 29 days, All 30 days	
Binge drinking	During the past 30 days, on how many days did you have 4 or more drinks of alcohol in a row (if you are female) or 5 or more drinks of alcohol in a row (if you are male)? "In a row" means within a couple of hours. 0 days, 1 day, 2 days, 3 to 5 days, 6 to 9 days, 10 to 19 days, 20 or more days	During the past 30 days, on how many days did you have 4 or more drinks of alcohol in a row (if you are female) or 5 or more drinks of alcohol in a row (if you are male)? "In a row" means within a couple of hours. 0 days, 1 day, 2 days, 3 to 5 days, 6 to 9 days, 10 to 19 days, 20 or more days	Any vs. none
Drunk	During the past 30 days, how many times have you been drunk from drinking alcoholic beverages? 0 times, 1 or 2 times, 3 to 5 times, 6 to 9 times, 10 to 19 times, 20 or more times	During the past 30 days, how many times have you been drunk from drinking alcoholic beverages? 0 times, 1 or 2 times, 3 to 5 times, 6 to 9 times, 10 to 19 times, 20 or more times	Any vs. none
Drinking alone	During the past 30 days, how many times did you drink alcohol by yourself? 0 times, 1 or 2 times, 3 to 5 times, 6 to 9 times, 10 to 19 times, 20 or more times	During the past 30 days, how many times did you drink alcohol by yourself? 0 times, 1 or 2 times, 3 to 5 times, 6 to 9 times, 10 to 19 times, 20 or more times	Any vs. none
Cigarette use	During the past 30 days, on how many days did you smoke cigarettes? 0 days, 1 or 2 days, 3 to 5 days, 6 to 9 days, 10 to 19 days, 20 to 29 days, All 30 days	During the past 30 days, on how many days did you smoke cigarettes? 0 days, 1 or 2 days, 3 to 5 days, 6 to 9 days, 10 to 19 days, 20 to 29 days, All 30 days	Any vs. none
Electronic nicotine delivery system (ENDS) use	A composite of 3 questions: These questions ask about vaping, which means using electronic vapor products, such as JUUL, SMOK, Suorin, Vuse, and blu. Electronic vapor products include e-cigarettes, vapes, vape pens, e-cigars, e-hookahs, hookah pens, and mods. They also include disposable products that you can only use once, such as Puff Bars, Stig, or Viigo. 1) During the past 30 days, on how many days did you use an electronic vapor product? 2) During the past 30 days, on how many days did you use an electronic vapor product on school property? 0 days, 1 or 2 days, 3 to 5 days, 6 to 9 days, 10 to 19 days, 20 to 29 days, All 30 days 3) During the past 30 days, on how many days did you use vapor products that contain nicotine (nicotine is a chemical in tobacco that can make you have cravings and become addicted)? 0 days, 1 or 2 days, 3 to 9 days, 10 to 19 days, 20 or more days	A composite of 3 questions: These questions ask about vaping nicotine using electronic vapor products, such as JUUL, Vuse, Logic, and MyBlu. Electronic vapor products include e-cigarettes, vapes, vape pens, e-cigars, e-hookahs, hookah pens, and mods that contain nicotine. They also include disposable products that you can only use once, such as Puff Bars Stig, Viigo, and Fruyt Stik. 1) During the past 30 days, on how many days did you use an electronic vapor product? 2) During the past 30 days, on how many days did you use an electronic vapor product on school property? 0 days, 1 or 2 days, 3 to 5 days, 6 to 9 days, 10 to 19 days, 20 to 29 days, All 30 days 3) During the past 30 days, on how many days did you use vapor products that contain nicotine (nicotine is a chemical in tobacco that can make you have cravings and become addicted)? 0 days, 1 or 2 days, 3 to 9 days, 10 to 19 days, 20 or more days	Any vs. none
Cannabis use	A composite score of 5 questions: 1) During the past 30 days, how many times did you use marijuana? 0 times, 1	A composite score of 5 questions: 1) During the past 30 days, how many times did you use marijuana? 0 times, 1	Any vs. none

	to 2 times, 3 to 5 times, 6 to 9 times, 10 to 19 times, 20 to 39 times, 40 or more times 2) During the past 30 days, how many times have you used marijuana in any of the following ways: smoked it (like in a joint or blunt)? 3) During the past 30 days, how many times have you used marijuana in any of the following ways: vaped it? 4) During the past 30 days, how many times have you used marijuana in any of the following ways: Ate or drank it in goods or products made with marijuana (This includes edibles, such as candy, baked goods, snacks, or drinks that contain marijuana.)? 5) During the past 30 days, how many times have you used marijuana in any of the following ways: used a concentrate or other high potency product (such as hash oil, dabs, wax, or shatter)? 0 times, 1 or 2 times, 3 to 9 times, 10 to 19 times, 20 to 39 times, 40 or more times	to 2 times, 3 to 5 times, 6 to 9 times, 10 to 19 times, 20 to 39 times, 40 or more times 2) During the past 30 days, how many times have you used marijuana in any of the following ways: smoked it (like in a joint or blunt)? 3) During the past 30 days, how many times have you used marijuana in any of the following ways: vaped it? 4) During the past 30 days, how many times have you used marijuana in any of the following ways: Ate or drank it in goods or products made with marijuana (This includes edibles, such as candy, baked goods, snacks, or drinks that contain marijuana.)? 5) During the past 30 days, how many times have you used marijuana in any of the following ways: used a concentrate or other high potency product (such as hash oil, dabs, wax, or shatter)? 0 times, 1 or 2 times, 3 to 9 times, 10 to 19 times, 20 to 39 times, 40 or more times	
**MENTAL HEALTH**			
Feel lonely	How often do you feel lonely? Never, Rarely, Sometimes, Often, Very often	How often do you feel lonely? Never, Rarely, Sometimes, Often, Very often	Mean score
Stress	During the past 30 days, how stressful has your life been? Not at all stressful, A little stressful, Somewhat stressful, Very stressful	During the past 30 days, how stressful has your life been? Not at all stressful, A little stressful, Somewhat stressful, Very stressful	Mean score
Anxiety symptoms: Generalized Anxiety Disorder 2-item (GAD-2)	Two items added together: 1) Over the last 2 weeks, how often have you been bothered by feeling nervous, anxious, or on edge? 2) Over the last 2 weeks, how often have you been bothered by not being able to stop or control worrying? 0:Not at all, 1:several days, 2:more than half the days, 3:nearly every day	Two items added together: 1) Over the last 2 weeks, how often have you been bothered by feeling nervous, anxious, or on edge? 2) Over the last 2 weeks, how often have you been bothered by not being able to stop or control worrying? 0:Not at all, 1:several days, 2:more than half the days, 3:nearly every day	Scores ≥ 3 vs. scores < 3
Depressive symptoms related to anxiety: Modified from Patient Health Questionnaire-9 (PHQ-9)	7-item scale: During the past two weeks, how often have you felt so stressed, anxious, or worried that you: 1) had little interest or pleasure in doing things? 2) had trouble falling asleep or staying asleep, or slept too much? 3) had felt tired or had little energy? 4) had a poor appetite or ate too much? 5) felt bad about yourself or that you were a failure or had let yourself or your family down? 6) had trouble concentrating on school? 7) had trouble concentrating on things outside of school, such as	7-item scale: During the past two weeks, how often have you felt so stressed, anxious, or worried that you: 1) had little interest or pleasure in doing things? 2) had trouble falling asleep or staying asleep, or slept too much? 3) had felt tired or had little energy? 4) had a poor appetite or ate too much? 5) felt bad about yourself or that you were a failure or had let yourself or your family down? 6) had trouble concentrating on school? 7) had trouble concentrating on things outside of school, such as	Summary score

	watching videos or shows or reading for pleasure? 0:Never, 1:Rarely, 2:Sometimes, 3:Often, 4:Very often	watching videos or shows or reading for pleasure? 0:Never, 1:Rarely, 2:Sometimes, 3:Often, 4:Very often	
Considered suicide	During the past 12 months, did you ever seriously consider attempting suicide? Yes, No	During the past 12 months, did you ever seriously consider attempting suicide? Yes, No	Any vs. none
Planned suicide	During the past 12 months, did you make a plan about how you would attempt suicide? Yes, No	During the past 12 months, did you make a plan about how you would attempt suicide? Yes, No	Any vs. none
Attempted suicide	During the past 12 months, how many times did you actually attempt suicide? 0 times, 1 time, 2 or 3 times, 4 or 5 times, 6 or more times	During the past 12 months, how many times did you actually attempt suicide? 0 times, 1 time, 2 or 3 times, 4 or 5 times, 6 or more times	Any vs. none
**STUDENTS' HELP-SEEKING AT SCHOOL**			
Can talk to adult at school if they have a problem	Is there at least one teacher or adult at your school that you can talk to if you have a problem? Yes, one; Yes, more than one; No, Not sure	Is there at least one teacher or adult at your school that you can talk to if you have a problem? Yes, one; Yes, more than one; No, Not sure	Yes vs. no
Mental health service use at school	A composite of 3 questions: Emotional challenges include things that make you feel sad, angry, stressed, or anxious, or that may make you have thoughts of hurting yourself. During the past 12 months, how many times did you talk with any of the following for help with emotional challenges or problems? 1) School counselor, school therapist, or school psychologist (Do not include talking about class scheduling or college or career preparation.) 2) School nurse 3) Another adult from school, like a teacher or other school staff. 0 times, 1 time, 2 or 3 times, 4 or more times	A composite of 3 questions: Emotional challenges include things that make you feel sad, angry, stressed, or anxious, or that may make you have thoughts of hurting yourself. During the past 12 months, how many times did you talk with any of the following for help with emotional challenges or problems? 1) School counselor, school therapist, or school psychologist (Do not include talking about class scheduling or college or career preparation.) 2) School nurse 3) Another adult from school, like a teacher or other school staff. 0 times, 1 time, 2 or 3 times, 4 or more times	Any vs. none
Barriers to seeking mental health services at school	A 5-item scale: How much do you agree or disagree with each statement about getting help from someone at school? 1) I don't know who to go to for help at school. 2) I don't think counseling with someone at school would help. 3) I don't trust anyone from my school enough to talk about my emotional challenges or problems. 4) A school counselor/therapist might not understand me or the challenges I was having. 5) Teachers or other school staff might treat me differently or give me fewer opportunities at school. Strongly agree, Agree, Neither agree nor disagree, Disagree, Strongly disagree	A 5-item scale: How much do you agree or disagree with each statement about getting help from someone at school? 1) I don't know who to go to for help at school. 2) I don't think counseling with someone at school would help. 3) I don't trust anyone from my school enough to talk about my emotional challenges or problems. 4) A school counselor/therapist might not understand me or the challenges I was having. 5) Teachers or other school staff might treat me differently or give me fewer opportunities at school. Strongly agree, Agree, Neither agree nor disagree, Disagree, Strongly disagree	Mean score

Talked to adult at school about being bullied	During the past 12 months, how many times did you talk to an adult from school about being bullied? Never, once, 2 or more times	During the past 12 months, how many times did you talk to an adult from school about being bullied? Never, once, 2 or more times	Any vs. none among those who reported being bullied at school
Talked to adult at school about being cyberbullied	During the past 12 months, how many times did you talk to an adult from school about being cyberbullied? Never, once, 2 or more times	During the past 12 months, how many times did you talk to an adult from school about being cyberbullied? Never, once, 2 or more times	Any vs. none among those who reported being cyberbullied

Table S3. These are the demographic and potential confounder questions asked in the staff and student surveys.

Construct	Staff Surveys	Student Surveys
Sexual identity	Which of the following best describes you? (Please mark all that apply.) Heterosexual (Straight); Gay or lesbian; Bisexual; Queer; Not sure; Another non-heterosexual identity, please specify: _______	2021 & 2023 MWAHS: Which of the following best describes you? Heterosexual (Straight); Gay or lesbian; Bisexual; I describe my sexual identity some other way; I am not sure about my sexual identity (questioning); I do not know what this question is asking.
Gender identity and sex assigned at birth	Which of the following best describes you? (Select one or more responses.) Woman; Man; Transgender woman; Transgender man; Genderqueer; Non-binary; Another identity, please specify: _______<br>What sex were you assigned at birth, on your original birth certificate? Male; Female	2021 MWAHS: A transgender person is someone whose biological sex at birth does not match the way they think or feel about themselves. Are you transgender? No, I am not transgender; Yes, I am transgender and I think of myself as really a boy or man; Yes, I am transgender and I think of myself as really a girl or woman; Yes, I am transgender and I think of myself in some other way; I do not know if I am transgender; I do not know what this question is asking.<br>2021 MWAHS: What is your sex? (This question is asking about your biological sex at birth, that is, what the doctor put on your birth certificate.) Female; Male<br>2023 MWAHS: Some people describe themselves as transgender when their sex at birth does not match the way they think or feel about their gender. Are you transgender? No, I am not transgender; Yes, I am transgender; I am not sure if I am transgender; I do not know what this question is asking.<br>2023 MWAHS: What sex were you assigned at birth, on your original birth certificate? Female; Male
Racial/ethnic identity	What is your race? (Select one or more responses.) American Indian or Alaska Native; Asian; Black or African American; Native Hawaiian or Pacific Islander; White; Another racial identity, please specify: _____________<br>Are you Hispanic or Latino? Yes; No	2021 and 2023 MWAHS: How do you describe yourself? Mark all that apply. American Indian or Alaska Native; Asian; Black or African American; Native Hawaiian or Other Pacific Islander; White; Other<br>2021 and 2023 MWAHS: Are you Hispanic or Latino/a? Yes; No
Age	What is your date of birth?	2021 and 2023 MWAHS: How old are you? 13 years old or younger; 14 years old; 15 years old; 16 years old; 17 years old, 18 years old or older
Role(s) in school	What is your role in the school? (Select one or more responses.) I do not work in a school; Teacher; Special education teacher; Administrator, please specify; Prevention staff nurse or health aide; Counselor or psychologist; Police, resource officer, or safety personnel; Paraprofessional, teacher assistant, or instructional aide; Other certified staff (e.g., librarian), please specify; Other	

	classified staff (e.g., janitorial, secretarial or clerical, food service), please specify; Something else, please specify
Years working as staff member	How many years have you worked at any primary or secondary school (K-12)? Open-ended
Highest education level achieved	What is the highest degree or level of school you have completed? No schooling completed; Nursery school; Grades 1 through 11; 12th grade-no diploma; Regular high school diploma; GED or alternative credential; Some college credit, but less than 1 year of college; 1 or more years of college credit, no degree; Associates degree (for example: AA, AS); Bachelor's degree (for example: BA, BS); Master's degree (for example: MA, MS, MEng, MEd, MSW, MBA); Professional degree beyond bachelor's degree (for example: MD, DDS, DVM, LLB, JD); Doctorate degree (for example, PhD, EdD)
Childhood religious affiliation	In what religion(s) were you raised? (Select one or more responses.) None; Catholicism; Judaism; Islam; Protestantism; Buddhism; Hinduism; Something else, please specify:______
Current religious affiliation	What religion(s) do you practice now? (Select one or more responses.) None; Catholicism; Judaism; Islam; Protestantism; Buddhism; Hinduism; Something else, please specify______
Religiosity	Duke University Religion Index: The following section contains 3 statements about religious belief or experience. Please mark the extent to which each statement is true or not true for you. 1) In my life, I experience the presence of the Divine (i.e., God). 2) My religious beliefs are what really lie behind my whole approach to life. 3) I try hard to carry my religion over into all other dealings of my life. Definitely true of me, Tends to be true, Unsure, Tends not to be true, Definitely not true
Religious attendance	Duke University Religion Index: How often do you attend religious meetings (e.g. church)? Never, Once a year or less, A few times a year, A few times a month, Once a week, More than once/week
Religious activities	Duke University Religion Index: How often do you spend time in private religious activities, such as prayer, meditation or Bible study? Rarely or never, A few times a month, Once a week, Two or more times/week, Daily, More than once a day

Past contact with LGBTQ+ people	These questions ask about your personal experiences. How many of your friends, relatives, or close acquaintances let you know that they were... 1) Gay or lesbian? 2) Bisexual? 3) Queer? 4) Transgender or another gender besides man/woman (e.g., genderqueer)? 5) Nonbinary or genderqueer? None; 1; 2 or 3; 4 or 5; 6 to 9; 10 or more
Past encounters working with LGBTQ+ youth	Indicate how much the following statements apply to you. 1) I have experience working with youth. 2) I have experience working with gay or lesbian youth. 3) I have experience working with bisexual youth. 4) I have experience working with queer youth. 5) I have experience working with transgender youth or youth who identify as another gender besides boy/girl (e.g., genderqueer). 6) I have experience working with genderqueer or nonbinary youth. I'm not sure; None; A little; Some; A great deal
Previous professional development related to working with LGBTQ+ youth	1) Have you participated in training about LGBTQ+ issues before? Yes; No; Not sure 2) If yes, approximately how many hours of training have you received? Open-ended numeral 3) Have you participated in eLearning or online training courses about LGBTQ+ issues before? Yes; No; Not sure 4) If yes, approximately how many hours of online training have you received? Open-ended numeral
Attitudes towards LGBTQ+ people	Modified Attitudes Toward Lesbians and Gay Men Scale: These questions ask about people with diverse sexual orientations and gender identities. Read each statement and select your level of agreement or disagreement. 1) Sex between two men is just plain wrong. 2) I think make homosexuals (gays) are disgusting). 3) Make homosexuality is a natural expression of sexuality in men. 4) Sex between two women is just plain wrong. 5) I think female homosexuals (lesbians) are disgusting. 6) Female homosexuality is a natural expression of sexuality in women. 7) Having sex with both males and females is just plain wrong. 8) I think bisexuals are disgusting. 9) Bisexuality is a natural expression of sexuality in people. 10) A person whose sex assigned at birth (male or female) does not match their gender identity is just plain wrong. 11) I think transgender people are disgusting. 12) Being transgender is a natural expression of gender identity. 13) I think gender non-conforming people are disgusting. Strongly agree,

	Somewhat agree, Neither agree nor disagree, Somewhat disagree, Strongly disagree	
Social desirability	Marlowe Crowne scale: 1) It is sometimes hard for me to go on with my work if I am not encouraged. 2) I sometimes feel resentful when I don’t get my way. 3) On a few occasions, I have given up doing something because I thought too little of my ability. 4) There have been times when I felt like rebelling against people in authority even though I knew they were right. 5) No matter who I’m taking to, I’m always a good listener. 6) There have been occasions when I took advantage of someone. 7) I’m always willing to admit to it when I make a mistake. 8) I sometimes try to get even rather than forgive and forget. 9) I am always courteous, even to people who are disagreeable. 10) I have never been irked when people expressed ideas very different from my own. 11) There have been times when I was quite jealous of the good fortune of others. 12) I am sometimes irritated by people who ask favors of me. 13) I have never deliberately said something that hurt someone’s feelings. True; False	
Grade In School		2021 and 2023 MWAHS: In what grade are you? 9th grade; 10th grade; 11th grade; 12th grade; Ungraded or other grade
Language spoken at home		2021 and 2023 MWAHS: What language is spoken most of the time in your home? English; Spanish; Portuguese; Another language
Acculturation		2021 and 2023 MWAHS: How long have you lived in the United States? Less than 1 year; 1 to 3 years, 4 to 6 years; More than 6 years, but not my whole life; I have always lived in the United States
Lifetime sexual abuse		2021 and 2023 MWAHS: During your life, has anyone ever had sexual contact with you against your will? Yes, once; Yes, more than once; No
Free/reduced price lunch status		2021 MWAHS: Are you eligible to get a free or reduced-price lunch at school? (Receiving free or reduced-price lunches means that lunch at school is provided to you for free or you pay less for it.) Yes; No; Not sure
