# Supplementary material for: Providing Lesbian, Gay, Bisexual, Transgender, Nonbinary, and Queer Adolescents With Nurturance, Trustworthiness, and Safety: Protocol for Pilot Cluster Randomized Controlled Trial Design
Source: JMIR Res Protoc. 2024 Mar 19;13:e55210. doi: 10.2196/55210 (PMC10988370; doi:10.2196/55210)
Supplement: Multimedia Appendix 2 [file resprot_v13i1e55210_app2.pdf]

1K01AA027564-01 Coulter, Robert

RESUME AND SUMMARY OF DISCUSSION: The candidate for this K01 award requests support for training in developing effective-learning interventions for substance use among sexual minority youth. The candidate was viewed as strong with a good track record of productivity and dissemination of on health-related information among sexual minority youth. The current mentoring team is exceptional and well matched with the candidates' goals. The career development plan was described as clear and well organized, albeit very ambitious. There is limited training in quantitative approaches targeted for small clusters. There were questions about feasibility and the need to get permission from schools prior to implementing the research, and the relatively distal pathway from intervening with educators to mediate changes in alcohol and drug use among individual youths. Overall, there was excellent enthusiasm for this application.

DESCRIPTION (provided by applicant): Alcohol and drug use (e.g., tobacco and marijuana use) are major public health problems affecting large proportions of youth. Alcohol and drug use also disproportionately burden certain youth populations, such as sexual minority youth (SMY; i.e., adolescents who identify as gay/lesbian or bisexual, or who have same- gender sexual behaviors or attractions). Compared with heterosexual youth, SMY have up to 600% higher odds of lifetime drug and alcohol use. Despite these substantial disparities, few evidence-based interventions exist for reducing alcohol and drug use among SMY. Nevertheless, SMY who report having supportive adults at school, greater school connectedness, and lower bullying victimization have lower drug and alcohol use. Therefore, an intervention that trains school staff (e.g., teachers, principals, nurses, counselors) to better understand SMY, support SMY, and engage in positive bystander behaviors that protect SMY from bullying victimization may reduce sexual-orientation disparities in drug and alcohol use. Furthermore, many school staff desire to support SMY, but they report a lack of training as their primary impediment. To address these gaps, I will execute two Specific Aims. In Aim #1, I will develop an online e-learning intervention aimed at improving school staff's knowledge, skills, and self-efficacy for supporting SMY. Using the intervention mapping approach, I will develop, user-test, and refine intervention materials by: conducting focus groups with a School Staff Advisory Board; performing usability tests via think aloud interviews with school staff; and collaborating with professional e-learning developers. In Aim #2, I will pilot test the feasibility and preliminary efficacy of this school staff-based intervention using a two-armed cluster-randomized controlled trial. I will enroll high schools participating in the fully-funded survey infrastructure of the MetroWest Adolescent Health Survey (MWAHS), located outside Boston, Massachusetts, where sexual-orientation disparities in drug and alcohol use still exist. Biennially, the MWAHS administers surveys to all students in each school, providing ample student-level data. In tandem, I will collect new longitudinal survey data (baseline and 6-month follow-up) from all school staff within each enrolled school. This e-learning intervention is easily modifiable and economically scalable, making it apt for wide dissemination with the potential for population-level impact in reducing drug and alcohol use disparities. To successfully complete my research, I will acquire training in: (1) the development of stakeholder- informed interventions, particularly e-learning programs; (2) implementation science research; and (3) the design and analysis of experimental studies. Guided by a strong interdisciplinary mentorship team, my planned training activities include mentorship meetings, formal coursework, training institutes, scientific seminars, and research conferences. Upon successful completion of my research and training plans, I will be propelled towards achieving my long-term goal, which is to become an independent scientific researcher specializing in stakeholder-informed intervention science aimed at reducing drug and alcohol use disparities for SMY.

PUBLIC HEALTH RELEVANCE

The Institute of Medicine's 2011 report titled "The Health of Lesbian, Gay, Bisexual, and Transgender (LGBT) People" calls for the development of evidence-based interventions for reducing sexual minority youth's large and persistent disparities in drug and alcohol use. The goals of the proposed research are to develop, and pilot test an e-learning intervention that trains school staff to better understand, support,

and protect sexual minority youth, and this novel intervention is hypothesized to increase adult support and school connectedness and simultaneously decrease bullying victimization, drug use, and alcohol use among sexual minority youth. Given the dearth of published research on alcohol and drug use interventions for sexual minority youth, this project will provide novel scientific contributions that can help foster health equity for sexual minority youth.

CRITIQUE 1

Candidate: 1
Career Development Plan/Career Goals /Plan to Provide Mentoring: 1
Research Plan: 2
Mentor(s), Co-Mentor(s), Consultant(s), Collaborator(s): 2
Environment Commitment to the Candidate: 1

Overall Impact:

This K01 application seeks mentored training and research support for Dr. Coulter, who's training to date has been primarily epidemiological in nature and whose research has focused quite consistently on health disparities among sexual minority youth. Under this award, the applicant would seek training in development of e-learning interventions, implementation science, and experimental designs (especially clinical trials), all while developing and pilot testing an e-learning intervention for high school staff promoting effective support of sexual minority youth. This application has many significant strengths, including a highly productive applicant (28 publications to date, 14 as first author), a uniquely well qualified primary mentor and team of advisors, a strong training plan, and a creative pilot trial concept. Concerns are relatively minor (letters of support from the targeted schools are not from top school administrators; there appear to be two primary hypotheses, each of which includes two outcomes; inadequate detail regarding prior intervention research in this area; need for more information on alcohol use disorders among sexual minority youth).

1. Candidate:

Strengths

- The applicant has been highly productive to date, with 28 publications (14 as first author). Potential to develop into an independent and productive investigator is clear.
- Letters of reference are highly supportive.
- Prior experience in the overall topic area is strong and augmented nicely by experience working with the biennial survey that will provide infrastructure and school-level data regarding student functioning.
- The applicant's prior work shows a laudable consistency in its focus on health disparities among SMY.
- The candidate's potential and proposed training around clinical trial management/analysis are excellent.

Weaknesses

- None noted.

2. Career Development Plan/Career Goals & Objectives/Plan to Provide Mentoring:

Strengths

- The plan for training in e-learning intervention development, implementation science, and skills in cluster randomized trials is sound and will contribute substantially to development of scientific independence.
- The training plan includes a strong array of relevant experiences including mentoring, formal coursework, training institutes, seminars, and conferences (e.g., a course on e-learning at Carnegie Mellon).
- Training activities make up an appropriate proportion (declining each year) of overall activities.
- Monitoring of research and training progress will be done by Dr. Miller, and also by (1) a bi-annual oversight committee including senior investigators with whom Dr. Coulter has previously worked; and (2) annual progress report evaluation from Dr. Coulter's Department Chairs.

Weaknesses

- Year one includes three courses as well as three training institutes, which appears overly ambitious given other proposed activities.
- Dr. Coulter's level of preparation and prior experience being mentored by Dr. Miller suggests that five years of support may not be needed.

3. Research Plan:

Strengths

- Sexual minority youth (SMY) have much higher lifetime rates of drug and alcohol use than heterosexual youth, and interventions to address this disparity are in short supply compared to interventions focused on disparities in HIV.
- Sexuality-based stigma and bullying victimization appear to be important drivers of health disparities among SMY.
- Existing interventions are often difficult to disseminate or implement.
- The PI already has IRB approval to do qualitative interviews with school staff on this issue, which supports overall feasibility.
- The proposed plan skillfully leverages an ongoing survey which would provide school-level data on participant functioning following the intervention.
- The proposal includes a strong plan for seeking input from stakeholders before development begins and after draft versions are available.
- Intervention design will leverage existing research regarding reduction of stigma around mental illness.

Weaknesses

- Clear evidence regarding disparities in the burden of alcohol use disorders among SMY (in addition to elevated use) would help in evaluating the larger significance of this application.
- Similarly, detail on existing interventions for SMY for substance use—or for HIV that include substance use and might be fruitfully adapted—is needed. For example, the applicant refers to an existing intervention in this area, with "minimal effects." Small effects are common and potentially important and may in fact be true for the proposed intervention.
- There appear to be two primary hypotheses, each of which appears to include two separate constructs (e.g., school staff will have high participation and low attrition; school staff will report high acceptability and show high demand).

- Acceptability will be measured via subsequent surveys to school staff, which is likely to result in missing data and reduced validity (presuming elapsed time between intervention and satisfaction survey).
- Schools must agree to participate in the study and must give permission for their MWAHS survey data to be provided to Dr. Coulter. Letters of support are provided from school nurses and wellness coordinators but not from top administrators, who ultimately must provide permission to participate.

4. Mentor(s), Co-Mentor(s), Consultant(s), Collaborator(s):

Strengths

- Primary mentor Dr. Miller is highly productive and has expertise in school-based interventions to reduce violence and improve health outcomes, using cluster-randomized trials.
- The advisory team has clear and complementary expertise in the areas targeted by the applicant (e.g., Dr. Abebe is a statistician with expertise in cluster-randomized trials; Dr. O'Donnell directs the survey that will be leveraged for this application; Dr. Norman is an expert in e-learning intervention design).

Weaknesses

- The applicant already has a substantial record of having worked with Drs. Mair and O'Donnell.

5. Environment and Institutional Commitment to the Candidate:

Strengths

- The University of Pittsburgh provides an ideal environment in which to conduct the proposed training and research.
- The letter from Department Chairs Drs. Albert and Dermody demonstrates strong support for an Assistant Professor position with protected time for activities under the proposed award.

Weaknesses

- None noted.

Protections for Human Subjects:

Acceptable Risks and Adequate Protections

- Human subject's plans are provided separately for Aims 1 and 2, and appropriately note and address the (minimal) possible risks. MWAHS data are school-level and anonymous.

Data and Safety Monitoring Plan (Applicable for Clinical Trials Only):

Acceptable

- o The applicant proposes an internal data safety and monitoring board consisting of himself and primary mentor Dr. Miller. Together they will monitor adverse events and data integrity.

Inclusion of Women, Minorities and Children:

- Sex/Gender: Distribution justified scientifically
- Race/Ethnicity: Distribution justified scientifically
- For NIH-Defined Phase III trials, Plans for valid design and analysis: Scientifically acceptable

- Inclusion/Exclusion of Children under 18: Including ages < 18; justified scientifically
- Child participants will accurately reflect the high schools from which they are drawn; in past surveys, approximately half of students were female and 80% were white. The applicant expects that school staff will be 65% female and 95% White.

Training in the Responsible Conduct of Research:

Acceptable

Comments on Format (Required):

- Training will take place via mentoring meetings, online training, RCR workshops, seminars, and an RCR course from the University of Pittsburgh.

Comments on Subject Matter (Required):

- Training will include identifying issues in RCR; author responsibilities; publication; data management; power; research collaborations; data security and confidentiality; ethical data interpretation; informed consent; conflicts of interest; and data sharing.

Comments on Faculty Participation (Required; not applicable for mid- and senior-career awards):

- The applicant's mentors will provide direct instruction during mentorship meetings and will include overall progress regarding RCR as part of progress reports.

Comments on Duration (Required):

- Duration for the ethics and RCR class = 32 hours over a 3-month period; this does not include other activities such as workshops, mentorship meetings, and online training.

Comments on Frequency (Required):

- Training in RCR will take place primarily in year 1, but also during each subsequent project year via mentorship meetings, biweekly adolescent and young adult research seminars, and teaching a course on RCR specifically with respect to SMY (designed in year 2 and taught each subsequent year).

Resource Sharing Plans:

Unacceptable

- None present.

Budget and Period of Support:

- Recommend as Requested

CRITIQUE 2

Candidate: 2
Career Development Plan/Career Goals /Plan to Provide Mentoring: 2
Research Plan: 4
Mentor(s), Co-Mentor(s), Consultant(s), Collaborator(s): 1
Environment Commitment to the Candidate: 1

Overall Impact:

This K01 proposal seeks to progress the candidate's career from epidemiologic researcher to intervention scientist. The proposed career development and training plan is focused on intervention development, implementation science, and experimental studies of intervention. The long-term goal of this proposal is for this candidate to become an independent scientific researcher whose work focuses on reducing drug and alcohol use inequities for sexual minority youth by developing, implementing, and testing interventions. The K01 research proposal is for an intervention science project that will develop an online e-learning intervention focused on improving the knowledge, skills, and self-efficacy of high school teachers, principals, nurses, and counselors in support of sexual minority youth. The project would then test intervention feasibility through participation, attrition, acceptability, and demand. It would additionally, using a 2-armed cluster-randomized controlled trial, provide a preliminary assessment of measurable effects through treatment group contrast to control group on outcomes of increased support and school connectedness, decreased bullying victimization and alcohol and drug use, and reduced sexual orientation disparities in alcohol and drug use. The candidate has an impressive training background in behavioral and community health sciences, with special emphasis on the epidemiology of alcohol and drug use among sexual minority youth. The candidate has been a NIDA F31 predoctoral fellow who conducted his dissertation on sexual-orientation disparities, a TL1 postdoctoral fellow, and has a track record of 28 articles in high impact peer-reviewed journals. He seems well prepared to do important work in this area, though could benefit from enhanced background in intervention work and intervention experience. The career development and mentoring plan is extremely well thought out and integrated. The ultimate goals of the candidate are elaborated into 3 tightly focused training areas, which focus further into very specific training objectives aligned to each career goal. The mentorship team is built around specific expertise addressing the training needs in one or more of each of these career objectives. The proposal would help fill a significant need for interventionists with clinical trials experience in the area of sexual minority health. Training focus is on intervention design, implementation science research, and design and analysis of cluster-randomized controlled trials, and a well-organized and highly detailed timeline specifies training activities. Areas of training that seem unfilled in the career development plan include strategies for research with difficult to reach populations, multi-level intervention, and small samples analysis. The research proposal is well conceived, highly detailed, and extremely well organized. Significance is found in its health disparities focus, as well as in how the intervention theory of change is based in observational data; this program of intervention science research will allow a controlled test of these relationships suggested from the observational literature. Innovation is highlighted in the way the research is informed by school stakeholders, and a strength of the proposed intervention is in how it is based in an existing model of change from the literature that enjoys considerable empirical support–the Information-Motivation-Behavioral Skills model. A clear measurement model with outcomes and potential confounds is specified, and the analysis section is thorough, detailed, and state of the art for intervention science. Four areas of weakness include first, how the mechanisms of action in the intervention are general protective factors for all populations, and are also protective of mental health, and the approach leaves unexplored other outcomes. This becomes potentially important risk may involve comorbidity of mental health concerns with alcohol, and risk reduction may occur through reduction in levels of other variables such as depression and anxiety in this population. Second, e-learning can no longer be described as highly innovative. Third, measurement approach is uneven; some of the variables are taped by a single item 'scale,' and in one case, this is despite a rich array of existing measures of the construct. Fourth, at least one of the tests of effects involves a three-way interaction without acknowledging the very limited power and potential instability of findings. The mentor team and environment supporting this K01 proposal are incredibly strong in their support of this applicant.

1. Candidate:

Strengths

- The candidate has had advanced graduate training in behavioral and community health sciences, with special emphasis on the epidemiology of alcohol and drug use among sexual minority youth.
- The candidate has been recipient of a NIDA F31 predoctoral fellowship to provide training in alcohol and drug use research, sexual minority youth health disparities, epidemiologic methods, and human development.
- The candidate's doctoral research focused on understanding sexual-orientation disparities in health by analyzing data from large epidemiologic studies.
- There is a respectable track record from this work of 28 articles in high impact peer-reviewed journals; most of these articles address health issues of sexual minority youth and several have been important and influential articles.
- As a National Center for Advancing Translational Sciences TL1 postdoctoral fellow, the candidate developed qualitative research skills, as well as organized community engagement activities including a stakeholder advisory group in a project preparatory to the K01 proposal research project.

Weaknesses

- The candidate describes limited background in clinical or human service work, which is often of great assistance to draw upon in intervention development activities; additionally, the candidate appears to have limited training background in complex intervention, also called community-level or multilevel intervention, which would be the type of intervention driving the rationale for the proposed cluster randomized trial analysis training.

2. Career Development Plan/Career Goals & Objectives/Plan to Provide Mentoring:

Strengths

- There is particular need for interventionists with clinical trials experience in the area of sexual minority health, and in this way, this proposed K01 pathway fills an important need.
- The candidate has developed a tight training focus on intervention design, implementation science research, and design and analysis of cluster-randomized controlled trials, and a specific career objective is aligned with each of these training goals.
- Each of the three career objectives is carefully laid in terms of specific training goals that then seamlessly integrate into specific activities under each career objective that are then associated with attendance at relevant training opportunities through mentorship meetings, coursework, training institutes, research conferences, and research group meetings.
- A well-organized and highly detailed timeline specifies training activities and research activities, and their linkages to the candidate's career objectives and proposed K01 research project specific aims.

Weaknesses

- The population of interest is often described in the literature as a ‘difficult to reach’ or ‘difficult to access’ population, and in addition to CBPR and community engaged research training, more general training in skills and methods associated with accessing these types of populations seems an important unaddressed area of training for this career trajectory.
- While the focus on cluster-based trials research seems appropriate, training in community-level or multi-level intervention, in contrast to individual focused intervention, appears warranted but

is not included; these types of intervention where the social unit is that of the group drive need to use cluster randomized trial research.

- In reviewing Table 5 and the characteristics of the 26 schools the proposed K01 study will randomize from, this proposed K01 study will involve small samples of sexual minority youth, and likely much of the candidate's future intervention science work in this area of research will work with comparatively small samples; yet, despite the strong mathematical background and interest of the candidate in inferential statistics, study of techniques and methods for small populations/samples analysis along with methods for difficult to access populations do not appear in the training curriculum and career development plan.

3. Research Plan:

Strengths

- The proposal is significant as it addresses sexual-orientation inequities in youth alcohol and drug use that are present even in U.S. states with low structural stigma, and sexual minority groups are a health disparities group for which intervention is an NIH priority.
- Significance is also found in the way the theory of change for the proposed intervention is based in observational research showing bullying victimization and stigma along with a lack of school connectedness and adult support at school contribute to sexual-orientation disparities in alcohol and drug use; from an intervention science perspective, this proposed program of research provides a test of these relationships.
- The proposal is innovative to the extent it is informed by school stakeholders composed of principal, teacher, school nurse, social worker, teacher training, and sexual minority young adult.
- Approach uses a detailed sequential model of behavioral intervention development based in the literature, and the proposed program of research is well aligned both with the candidate's previous work and the candidate's current NIH career pipeline.
- A strength of the proposed intervention is it is based in an existing model of change from the literature that enjoys considerable empirical support–the Information-Motivation-Behavioral Skills model, and accordingly, the intervention's objectives are to increase school teachers, principals, nurses, and counselors knowledge, self-efficacy, and use of sexual minority youth inclusive practices; knowledge and positive attitudes; active empathetic listening skills; and positive bystander behaviors when witnessing sexual minority related violence and aggression–this follows from the Information-Motivation-Behavioral Skills model where knowledge, motivation, and behavioral skills determine enactment of specific behaviors, in this case, behaviors that support sexual minority youth.
- The approach also includes a clear measurement model linking intervention components to intermediate variable staff outcomes, that in turn potentiate the ultimate variable youth outcomes.
- Proposed outcomes are thoroughly specified in Table 6, along with potential confounds that will be explored in the analyses.
- Analysis are detailed and very clearly described, well thought out, and each proposed analysis appears appropriate.

Weaknesses

- In addition to their potential role in reduction of alcohol and drug use, education to combat bullying victimization and stigma, and school connectedness and adult support at school are also general protective factors for both sexual minority and non-minority students, as well as lead to other beneficial outcomes including prevention of suicide and mental health disorders;

this literature is not considered, and effects in these areas is not considered. Why this is important is the effects on alcohol and drug use may co-occur with and may be mediated by mental health status such as decreased depressive/anxiety symptoms.

- At this point in time, e-learning can no longer be accurately described as a highly novel method for training, given its wide use across multiple disciplines and training areas; further, the assertion that a single training model would be culturally and regionally equivalent across all regions in the United States is high questionable, and in the area of support for sexual minority youth, in some cases may potentially be culturally inappropriate or even iatrogenic across distinct rural and cultural groups.
- Approach with regard to measurement is uneven: While some of the outcomes are sophisticated (ELSS/TBIMTBC, social connectedness) other outcome 'measures' such as school-based support, are a single item, in this case adapted from the YRBS; this is regrettable as this construct in particular has a long history of extensive work in the measurement of social support.
- While this is a feasibility study whose goals are to assess if the intervention is implementable and produces effects, and is not a test of efficacy, the final proposed test of secondary hypotheses with youth will require exploration of a three-way interaction term; given suggested sample size from Table 5, effect size estimates may not prove stable for the test of reduction of disparity.

4. Mentor(s), Co-Mentor(s), Consultant(s), Collaborator(s):

Strengths

- The interdisciplinary mentorship team was created with the specific goal and is ideally suited to progress the candidate's development in the K01 proposal's three career objectives and their corresponding training activities, the mentors seamlessly mesh to create this comprehensive training plan.
- Dr. Miller as primary mentor has training in medical anthropology as well as Internal Medicine and Pediatrics and is a mixed-methods intervention researcher trained in community-based participatory research with over 165 peer-reviewed publications. She is Director of Adolescent and Young Adult Medicine at Children's Hospital of Pittsburgh and Director of the Community Engagement Core (Community PARTners) of the Clinical and Translational Science Institute of the University of Pittsburgh; both entities can provide important support for this proposed K01 project. Particularly relevant to this K01 application, she has expertise in designing, implementing, and evaluating stakeholder-informed and school-based interventions with youth.
- Dr. Abebe is a biostatistician with significant clinical trials experience through leading statistical cores and data coordinating centers on a number of trials in various areas; Dr. Abebe also has considerable experience collaborating with early career investigators on career development awards. Dr. Abebe currently teaches clinical trials courses at the University of Pittsburgh and has specialized expertise in design and analysis of cluster-randomized controlled trials, an important need in the field of intervention science and for the K01 career development goals of this application.
- Dr. O'Donnell has a long history of important research with over 100 peer-reviewed publications largely in work with health care providers to understand AIDS stigma and improve the quality of health and social services, with important contributions to interventions in this area. She also has expertise and can provide training and guidance to the candidate in development and implementation of the proposed e-learning program.
- Dr. Norman is Director of the Innovative Design for Education and Assessment Lab at the University of Pittsburgh's Institute for Clinical Research Education, where he focuses on

developing innovative educational methods. His expertise, experience, and resources in developing and implementing online programs will assist Dr. Coulter as he designs his online e-learning training modules.

- Dr. Mair is an NIAAA-funded researcher who examines the social ecology of alcohol use and is Component Director of a recently funded P60 using agent-based modeling to study social ecological mechanisms underlie alcohol etiology in communities. She also has expertise in alcohol and drug use measurement and analysis and will provide training to the candidate in this area; additionally, she will provide intradepartmental mentorship to Dr. Coulter's primary faculty appointment as he shares with her an appointment in the Department of Behavioral and Community Health Sciences.

Weaknesses

- None noted.

5. Environment and Institutional Commitment to the Candidate:

Strengths

- University of Pittsburgh Graduate School of Public Health and School of Medicine have successful records of career development, and Dr. Coulter has appointments in the Department of Behavioral and Community Health Sciences and the Division of Adolescent and Young Adult Medicine.
- Department of Behavioral and Community Health Sciences has a strong reputation for community-based participatory research strategies.
- The Division of Adolescent and Young Adult Medicine also includes an interdisciplinary team of researchers. Notably, the Division of Adolescent and Young Adult Medicine supports a Youth Research Advisory Board. This board meets monthly and provides input on clinical and community-based research activities where youth members review research protocols, recruitment strategies and flyers, consent forms, literacy level, and dissemination materials. A Parent Advisory Group provides similar feedback on an ad hoc basis. This board and group have strong potential to be an important asset in the proposed K01 research project's implementation.
- IT support at this institution is exemplary, and includes a Technology Help Desk is available 24 hours a day, 7 days a week to answer scholars' technology-related questions
- Notably, Dr. Coulter will have access to the computing services offered through the Center for Research on Health Care Data Center. The center is directed by Dr. Kaleab Abebe, who is one of Dr. Coulter's mentors in this proposal. Center faculty and staff members provide statistical analysis, database development, website design, and other graphic design services to over 500 research projects.
- Dr. Coulter will have access to all resources of the Center for LGBT Health Research. The center is the first higher education institution for LGBT health research in the U.S., and it provides a formalized mentorship program for doctoral students, MPH students, and early career faculty, including Dr. Coulter.
- The University of Pittsburgh CTSI can provide an enormous range of services critical to the success of a K01 research project; these include access to services at the Responsible Conduct of Research Center that navigates all necessary regulatory pathways at any stage of a research projects, the Biostatistics, Epidemiology and Research Design Core that provides up to ten hours of support per project is provided at no cost and is available for grant application development, study design, data analysis, and data management consultation, the Regulatory Knowledge and Support Center (RKSC) Core, which assists in navigation of all necessary regulatory pathways, and the Community PARTners (Partnering to Assist Research and

Translation) Core, which focuses on community engagement support to develop research-informed community members and health professionals, as well as community-informed researchers.

Weaknesses

- None noted.

Protections for Human Subjects:

- Protections for human subjects is helpfully broken down by aim. The protections section includes careful consideration of risks, steps to mitigate risks, and benefits/knowledge to be gained, and appears to address all considerations necessary.

Data and Safety Monitoring Plan (Applicable for Clinical Trials Only):

Acceptable

- o The DSMB proposed is an Internal Data Safety and Monitoring Board (DSMB) will be comprised of the Principal Investigator (Dr. Coulter) and the Primary Mentor (Dr. Miller). As the trial is not blinded and low-risk, the PI has concluded it was appropriate to have the PI and Primary Mentor carry out the DSMB functions.

Inclusion of Women, Minorities and Children:

- Sex/Gender: Distribution justified scientifically
- Race/Ethnicity: Distribution justified scientifically
- For NIH-Defined Phase III trials, Plans for valid design and analysis: Scientifically acceptable
- Inclusion/Exclusion of Children under 18: Including ages < 18; justified scientifically
- Study will draw from gender and race/ethnicity proportionate to teaching faculty and staff ratios in Aim 1, and high school student ratios in Aim 2, and Aim 2 will be with high school students and include children under 18.

Training in the Responsible Conduct of Research:

Acceptable

Comments on Format (Required):

- Format will include discussions at mentorship meetings and research seminars, and didactic instruction at workshops, as well as development and presentation of a workshop on ethics in the topic area of this K01.

Comments on Subject Matter (Required):

- Subject matter will include mentorship meetings, CITI training, RCR Workshops organized by The University of Pittsburgh's Clinical and Translational Science Institute's (CTSI) RCR Training Center, completion of the University of Pittsburgh's Institute for Clinical Research Education "Ethnics and Responsible Conduct of Research" course, Attendance at Biweekly University of Pittsburgh School of Medicine Department of Pediatrics Adolescent and Young Adult Research Seminars, and very impressively, the Creation and Implementation of an RCR Workshop through The University of Pittsburgh's Clinical and Translational Science Institute's (CTSI) RCR Training Center entitled "Examining Sexual Orientation in Research: From Conceptualization to Dissemination of Results."

Comments on Faculty Participation (Required; not applicable for mid- and senior-career awards):

- Faculty will participate in the mentorship meetings and also in the development of the workshop on ethics in the topic area of this K01 entitled "Examining Sexual Orientation in Research: From Conceptualization to Dissemination of Results."

Comments on Duration (Required):

- Duration of mentorship activities are all delineated in Table 2, and are 1 hour, CITI is 2 hours, RCR Workshops are 8 workshops, the course is 32 hours and seminars are likely one hour, while the new workshop to be created is one hour, with significant preparatory time.

Comments on Frequency (Required):

- Frequency of mentorship activities are all delineated in Table 2, and are weekly or bimonthly, CITI is every 2 years, RCR Workshops are 8 workshops, the course is 32 hours, and seminars are biweekly, while the new workshop to be created is years 2-5.

Resource Sharing Plans:

Unacceptable

- No resource sharing plan or declaration of no relevant resources to share

Budget and Period of Support:

- Recommend as Requested

CRITIQUE 3

Candidate: 1
Career Development Plan/Career Goals /Plan to Provide Mentoring: 3
Research Plan: 4
Mentor(s), Co-Mentor(s), Consultant(s), Collaborator(s): 2
Environment Commitment to the Candidate: 1

Overall Impact:

Dr. Coulter has developed a comprehensive training plan to assist with the transition from epidemiologist studying sexual minority health to an applied interventionist reducing sexual minority-related disparities in alcohol and drug use. At the University of Pittsburgh, Dr. Coulter has assembled a team including primary mentor Miller and an advisory committee with complementary expertise that are well-suited to supervise this endeavor. The K01 will culminate in a small cluster RCT of a school-based staff intervention to improve the support of SMY in schools, with the ultimate outcome of attempting to reduce SMY disparities in drug use. The proposal is consistent with Dr. Coulter's prior experiences and future goals. It is also a quite ambitious plan in light of the core areas of training that would be provided in the first year (i.e. responsible conduct of research, implementation science, e-learning and intervention, clinical trial development and analysis).

1. Candidate:

Strengths

- The candidate has a strong track record of success in the field of health among sexual minority youth. The proposed training and project would directly build upon this prior work. Notably, Dr. Coulter has already published more than 28 articles, including several in the top addiction journals, and received an F31.

- The candidate has been provided exceptionally strong reference letters, each of which note the candidate’s ability to conduct independent research in this field and their enthusiasm for having Dr. Coulter as a colleague. Each letter supports Dr. Coulter’s high potential for becoming an independent investigator.
- Dr. Coulter appears dedicated to becoming an independent scholar in the field of reducing alcohol and drug use disparities for SMY.

Weaknesses

- The extent of Dr. Coulter’s prior experiences with original data collection in this area appears limited. Prior work has primarily focused on analyzing preexisting data from large epidemiologic studies.

2. Career Development Plan/Career Goals & Objectives/Plan to Provide Mentoring:

Strengths

- The clinical trial experience will clearly contribute to the applicant’s research career. The applicant has conducted epidemiological and mechanistic research in this area; whereas the clinical trial will address some of the very factors of concern that was identified in the prior research.
- Training will add to the candidate’s prior training and experiences, and will include areas of intervention development, implementation science, and design and analysis of experimental studies.
- Coulter’s progress will be monitored with annual evaluations and summarized in a progress report to department chairs.

Weaknesses

- It seems ambitious to be fully trained in intervention development, implementation science, and e-learning design principles and methods in order to implement/complete a clinical trial using these methods by year 3.

3. Research Plan:

Strengths

- The plan includes developing and evaluating an online e-learning intervention to train school staff to support SMY, which will meet unmet needs of staff to better support SMY and would be more easily scalable than an in-person intervention.
- There is a strong scientific premise for school-based interventions to reduce SMY drug use.
- The intervention will be developed and evaluated using rigorous methods, including intervention mapping process for development and pilot tested using a cluster-based RCT for evaluation.
- There is a clear path for this research plan developing into an R01 application.

Weaknesses

- Study is not measuring individual-level changes in drug use.
- It is not clear that 4 schools will be enough to obtain desired confidence interval estimates for individual-level change, in light of not having individual-level data on change in drug use.
- The scientific premise for addressing SMY stigma among staff to reduce sexual minority drug use disparities is relatively weak. The intervention does not address a key factor that likely plays a role in SMY drug use, which is peer use.
- It appears that gender identity is not accessed by MWAHS

- It is not clear if information on sexual orientation or other demographic factors will be collected from staff, to better understand the characteristics of individuals who choose to participate in the training
- High participation rates, low participation rates, and high demand and acceptability are predicted; however, the basis of these hypothesis are not clear.
- The nature of the control intervention/materials is not clear. It seems important to establish whether any SMY-related information would be provided. For instance, it is not clear if students will be aware of the training that staff would receive, and if simply having knowledge of the training occurring would alter perceptions of support (as opposed to the intervention changing staff behavior and creating an effect).

4. Mentor(s), Co-Mentor(s), Consultant(s), Collaborator(s):

Strengths

- Mentor Miller is highly qualified to provide training in clinical trials and intervention research in the field of alcohol use with community and school-based partners.
- Abebe is a PhD biostatistician and director of the Center for Clinical Trials & Data Coordination at the University of Pittsburgh and is well-suited to support Dr. Coulter to analyze this type of data.
- Additional advisory committee mentors will provide guidance on substance use interventions, school initiatives, and online technology and intervention approaches.

Weaknesses

- Mentorship team have limited experiences with sexual minority-focused interventions, which would have unique challenges and focal areas compared to substance use interventions for youth in general.

5. Environment and Institutional Commitment to the Candidate:

Strengths

- University of Pittsburgh appears committed to Dr. Coulter's career development, and the institution has provided Dr. Coulter with adequate research facilities and resources to conduct the proposed research and training.
- Dr. Coulter is being supported by two departments with a primary appointment in the Dept of Behavioral and Community Health Sciences and secondary in the Dept of Pediatrics.

Weaknesses

- None noted.

Protections for Human Subjects:

Acceptable Risks and Adequate Protections

- IRB approval has been obtained and data collection is ready to begin.

Data and Safety Monitoring Plan (Applicable for Clinical Trials Only):

Acceptable

Inclusion of Women, Minorities and Children:

- Sex/Gender: Distribution justified scientifically
- Race/Ethnicity: Distribution justified scientifically
- For NIH-Defined Phase III trials, Plans for valid design and analysis: Not applicable
- Inclusion/Exclusion of Children under 18: Excluding ages <18; justified scientifically

Training in the Responsible Conduct of Research:

Acceptable

Comments on Format (Required):

- Format is comprehensive, including mentorship meetings on RCR, completion of CITI modules, attendance in RCR workshops at the University of Pittsburgh, and biweekly research seminars

Comments on Subject Matter (Required):

- content is appropriately general for behavioral research as well as focused on clinical trial research

Comments on Faculty Participation (Required; not applicable for mid- and senior-career awards):

- Faculty will be involved in training as part of mentorship meetings

Comments on Duration (Required):

- Training will be appropriately focused in year 1 of the K01, but will continue for the duration of the project to a lesser extent

Comments on Frequency (Required):

- The frequency appears appropriate given the scope of training needed and the research being conducted.

Resource Sharing Plans:

- Not Included

Budget and Period of Support:

- Recommend as Requested

THE FOLLOWING SECTIONS WERE PREPARED BY THE SCIENTIFIC REVIEW OFFICER TO SUMMARIZE THE OUTCOME OF DISCUSSIONS OF THE REVIEW COMMITTEE, OR REVIEWERS' WRITTEN CRITIQUES, ON THE FOLLOWING ISSUES:

PROTECTION OF HUMAN SUBJECTS: ACCEPTABLE

INCLUSION OF WOMEN PLAN: ACCEPTABLE

INCLUSION OF MINORITIES PLAN: ACCEPTABLE

INCLUSION OF CHILDREN PLAN: ACCEPTABLE

COMMITTEE BUDGET RECOMMENDATIONS: The budget was recommended as requested.

Footnotes for 1 K01 AA027564-01; PI Name: Coulter, Robert William Stewart

NIH has modified its policy regarding the receipt of resubmissions (amended applications). See Guide Notice NOT-OD-14-074 at http://grants.nih.gov/grants/guide/notice-files/NOT-OD-14-074.html. The impact/priority score is calculated after discussion of an application by averaging the overall scores (1-9) given by all voting reviewers on the committee and multiplying by 10. The criterion scores are submitted prior to the meeting by the individual reviewers assigned to an application, and are not discussed specifically at the review meeting or calculated into the overall impact score. Some applications also receive a percentile ranking. For details on the review process, see http://grants.nih.gov/grants/peer_review_process.htm#scoring.
